# Rapa Nui (Easter Island) monument (*ahu*) locations explained by freshwater sources

**DOI:** 10.1371/journal.pone.0210409

**Published:** 2019-01-10

**Authors:** Robert J. DiNapoli, Carl P. Lipo, Tanya Brosnan, Terry L. Hunt, Sean Hixon, Alex E. Morrison, Matthew Becker

**Affiliations:** 1 Department of Anthropology, University of Oregon, Eugene, OR, United States of America; 2 Department of Anthropology and Environmental Studies Program, Binghamton University, Binghamton, NY, United States of America; 3 Department of Geological Sciences, California State University, Long Beach, Long Beach, CA, United States of America; 4 Honors College and School of Anthropology, University of Arizona, Tucson, AZ, United States of America; 5 Department of Anthropology, Pennsylvania State University, University Park, PA, United States of America; 6 International Archaeological Research Institute, Inc., Honolulu, HI, United States of America; 7 Department of Anthropology, University of Auckland, Auckland, New Zealand; New York State Museum, UNITED STATES

## Abstract

Explaining the processes underlying the emergence of monument construction is a major theme in contemporary anthropological archaeology, and recent studies have employed spatially-explicit modeling to explain these patterns. Rapa Nui (Easter Island, Chile) is famous for its elaborate ritual architecture, particularly numerous monumental platforms (*ahu*) and statuary (*moai*). To date, however, we lack explicit modeling to explain spatial and temporal aspects of monument construction. Here, we use spatially-explicit point-process modeling to explore the potential relations between *ahu* construction locations and subsistence resources, namely, rock mulch agricultural gardens, marine resources, and freshwater sources—the three most critical resources on Rapa Nui. Through these analyses, we demonstrate the central importance of coastal freshwater seeps for precontact populations. Our results suggest that *ahu* locations are most parsimoniously explained by distance from freshwater sources, in particular coastal seeps, with important implications for community formation and inter-community competition in precontact times.

## Introduction

Explaining the temporal and spatial patterns of monument construction as they relate to social complexity is a ‘grand challenge’ for contemporary archaeology [1–3]. Despite considerable research on this subject, formal analyses of the role that environmental factors play in the emergence of monument construction have been largely underdeveloped. Recent studies, however, have begun to employ spatially explicit modeling to explore how distributions of resources relate to monuments (e.g., [1,4–6]). These studies provide key insights into the degree to which ecological constraints shape the location and function of monuments in past societies.

**Fig 1:**
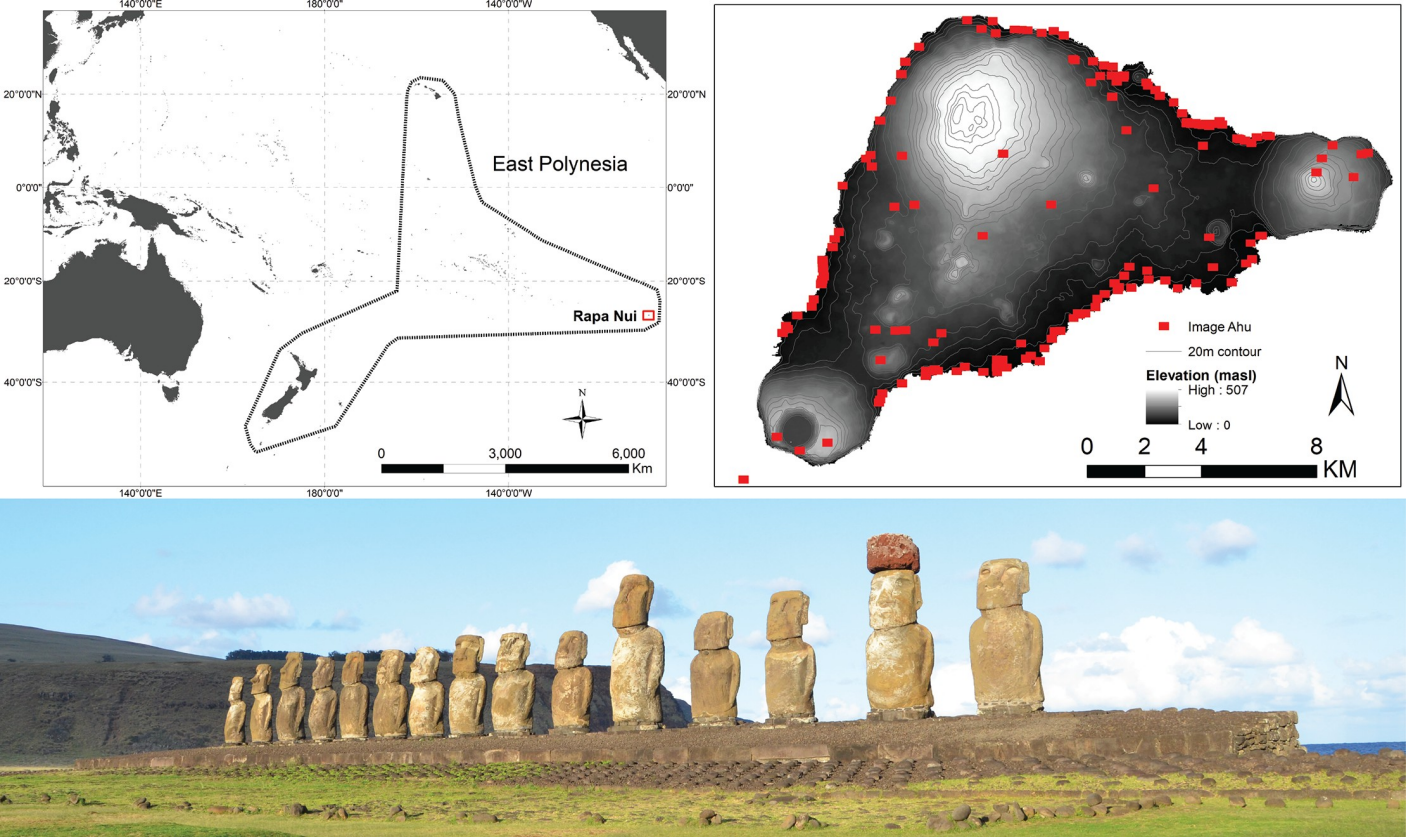
Rapa Nui. (Top left) Rapa Nui in East Polynesia, (top right) locations of image-*ahu* on Rapa Nui, and (bottom) *Ahu Tongariki* with *moai* (Photo by R.J. DiNapoli).

Rapa Nui (Easter Island, Chile, Fig 1) provides one of the most dramatic cases of prehistoric monument construction where, in a span of only about 500 years, from the 13^th^ century AD to European contact in AD 1722 and into historic times, the islanders (Rapanui) constructed over 300 megalithic platforms (*ahu*) and nearly 1000 multi-ton anthropomorphic statues (*moai*) [7–11]. The achievements of the Rapanui are even more impressive when one considers the island’s ecological marginality, including low and unpredictable rainfall, nutrient-poor soils, lack of large coral reefs or abundant sources of surface freshwater [12]. The island’s ecology greatly constrained the range of options available for subsistence to the island’s inhabitants [8,13], and many consider these environmental constraints to be a key factor in the emergence of monuments on Rapa Nui, such as their role as adaptive responses to environmental uncertainty (e.g., [14,15]) or as territorial signals of control over limited resources (e.g., [8,16–20]).

The relationship between the island’s subsistence resources and temporal and spatial patterns of monument construction, however, remain largely untested, which represents a significant limitation in our understanding of Rapa Nui’s history. Yet, like other oceanic islands, Rapa Nui can offer a model system for understanding human-environment interactions, including the ecological factors underlying monument construction [17,21–23]. Accordingly, the current work is concerned with beginning to test the hypothesis that Rapa Nui’s monumental architecture served as territorial signals of control over subsistence resources. As a starting point for evaluating this hypothesis, we quantitatively model how the spatial distribution of *ahu* is explained by different resources thought to be the focus of competition in precontact times. Tests are conducted through spatially-explicit modeling of the relations between *ahu* and the three critical subsistence resources on Rapa Nui: rock mulch agricultural gardens, marine resource locations, and freshwater sources. The expectation is that if Rapa Nui’s monuments were built, in part, to signal territorial resource control, then there should be a spatial association between *ahu* and the resources they are signaling control over [17]. In our work, we follow an information criteria model-selection approach using point-process models [24–27]. Our analyses combine existing data for the coverage of agricultural fields [28], marine resource locations, and include data from our ongoing study of the island’s freshwater sources [29,30]. For the purposes of this study, we restrict our spatial analysis of *ahu* to the eastern portion of the island where we have near-complete coverage documenting the distribution of these three resources.

The results of our point-process modeling indicate that the spatial locations of *ahu* are most parsimoniously explained by proximity to freshwater sources. Our findings offer an explanation for the primarily coastal distribution of these monuments as well as for *ahu* found inland. These results provide key information on the conditions that contributed to the unprecedented investments in monument construction on Rapa Nui.

## Background to Rapa Nui and its monuments

### Rapa Nui environment

Rapa Nui is a small (164 km^2^), isolated island in the southeastern Pacific that is about 3500 km from South America and nearly 2000 km from the nearest inhabited island (Fig 1). The island is volcanic in origin with three main shield volcanoes (Rano Kau, Terevaka, and Poike) and a number of smaller scoria and cinder cones [31]. With Terevaka at just over 500 meters above sea-level (MASL), the island is relatively low-lying and lacks incised valleys common on other Pacific islands. Rapa Nui’s climate is seasonal and windy, given its subtropical latitude, and it receives relatively low and unpredictable annual rainfall ranging from ca. 600–2000 mm/year [13,32]. While paleoecological studies demonstrate a once extensive palm forest (e.g., [33–36]), over the period of human occupation the island lost its forest with the combined effects of human land-clearing for cultivation and the invasive commensal Pacific rat (*Rattus exulans*) [37–40]. Compared to elsewhere in Polynesia, Rapa Nui’s soils are excessively-drained, leached, and poor in available nutrients [13,41,42]. Although there are freshwater lakes within the volcanic craters, given the very porous nature of the underlying substrate [43], the island lacks other sources of surface freshwater, such as permanent streams, common on other islands. As it rises steeply from the ocean floor, Rapa Nui also has a relatively impoverished marine environment and lacks large coral reefs or a lagoon [44]. These environmental characteristics imposed considerable constraints on the subsistence options available to precontact inhabitants.

### Rapa Nui’s monuments

The inherent environmental constraints make the achievements of human populations who persisted on this small island for more than 500 years all the more remarkable. Not only did Rapanui people manage to live successfully in a small, resource-poor, and isolated location, they collectively manufactured nearly one thousand massive stone statues (*moai*) and more than 300 megalithic platforms (*ahu*).

While a precise overall chronology for Rapa Nui’s monuments has not been fully established, radiocarbon dating indicates that construction of the island’s megalithic platforms, known collectively as *ahu*, likely began shortly after colonization in the 13^th^ century and intensified over time [7,45–49]. *Ahu* represent a derived form of ritual architecture found elsewhere in East Polynesia [10,50–52], though both the quantity and magnitude of investment on Rapa Nui are distinct. While many *moai* (ca. 400) remain at the statue quarry at Rano Raraku, hundreds of *moai*, weighing several tons each, were transported along statue roads and erected upon *ahu* [53–55]. In addition, many *moai* were also adorned with large red-scoria ‘hats’ called *pukao* [56,57]. Rectangular platform *ahu* with a dressed-stone sea-wall, and which often support one or more *moai*, are referred to as “image-*ahu*” [10]. Image-*ahu* are precontact features, whereas other *ahu* forms, such as semi-pyramidal *ahu*, *ahu po‘e po‘e*, and *ahu avanga* are considered largely post-contact in age [10]. Here, we focus our discussion and analyses on the distribution of image-*ahu* (Fig 1).

Image-*ahu* were the focal points of Rapa Nui’s precontact communities [10,47,58]. Overall, the Rapanui settlement pattern is characterized by relatively dispersed communities distributed along the coastline as redundant sets of domestic features ca. 100–200 m inland from *ahu* [19,59,60]. While image-*ahu* have a primarily coastal distribution and, based on limited historical accounts, were community gathering locations for ritual activity, explanations for why they occur in their specific locations or additional social roles these monuments may have served remain largely unanswered.

Many have debated whether *ahu* served as territorial displays of control or hereditary ownership over the island’s limited subsistence resources (e.g., [11,19,20,52,58,61–65]), and while there is general agreement that competition and territoriality were centered around limited and predictable resources (see [66]), there is disagreement over which resources were most critical. Van Tilburg [11] has argued that, “the archaeological evidence illustrates clearly that the control of subsistence production in agriculture and marine resources was intimately and strongly linked to the typical Polynesian scheme of hereditary land use rights…The need constantly to restate that ownership, generation after generation and in the context of a growing population and a changing natural environment, seems to have been one of the driving forces of *ahu* construction, although other social and religious motivations obviously existed.” Similarly, Kirch [18] has argued that image-*ahu*, “are found at the best embayments around the island,” which served as a means of, “visually ‘controlling’ access to the limited marine resources.” Others (e.g., [8,17,19]) suggest that freshwater would have been a significant critical limited resource and the focus of intense competition, whereby *ahu* served as costly signals of community competitive ability. In addition, many assume that surplus sweet potato yields from lithic mulch gardens would have been necessary to support *ahu* construction and *moai* transport and that monuments served to broadcast elite control of these resources (e.g., [20,67–70]). Stevenson and colleagues [67–70] have consistently argued for an association between *ahu* and rock mulch gardens, that the development of intensified agriculture closely tracks the tempo of monument construction, and that “[t]hese repeated associations indicate that ranked persons were most likely ritually involved with agricultural production and used the position to manage the field systems under their authority” [67]. To date, however, rigorous tests of these hypotheses are lacking, as are any attempts to construct formal models of *ahu* spatial patterns (but see Beardsley [71] for exploratory analyses). If image-*ahu* served as territorial markers of control over subsistence resources, as a majority of archaeologists who have worked on the island suggest, then the empirical expectation is a spatial association between *ahu* and the resources for which they mark control.

### Subsistence resources

Access to raw materials for architectural construction and tools seems to be unrelated to image-*ahu* locations. Obsidian used for cutting and scraping tools known as *mata‘a* [72–75] was derived from several discrete locations near their source volcanic vents away from *ahu* activity [76,77]. Basalt, on the other hand, which was used to create adzes and other tools, had many sources around the island, but there is no clear pattern suggesting localized control [78,79]. Stone for large *moai* and red scoria for *pukao* come primarily from single quarries at Rano Raraku and Puna Pau (respectively), and there do not appear to have been limits to the access of these materials by any particular group (e.g., [80–82]). Thus, on Rapa Nui there are three broad classes of resources to consider that might potentially relate to the choices made for constructing image-*ahu*: locations suitable for agriculture, sources of marine food, and freshwater.

#### Agriculture

Like Polynesians across the Pacific, the precontact Rapanui were agriculturalists, although, overall, Rapa Nui’s growing conditions are considered marginal when compared to elsewhere [13,42]. A substantial fraction of subsistence, however, depended upon agricultural crops that included sweet potato (*Ipomoea batatas*), yams (*Dioscorea alata*), dryland taro (*Colocasia esculenta*), bananas (*Musa* sp.), sugar cane (*Saccharum officianarum*), and other cultigens. Of these, plant microfossil analyses of soils and human dental calculus indicate that sweet potato was the primary plant food source [37,83,84]. The island’s cool climate and lack of streams or incised valleys meant that irrigated taro cultivation common elsewhere in Polynesia was not possible [85]. Instead, sweet potato, along with yams and dryland taro, were grown in lithic mulch gardens. In these gardens, a collection of basalt pebbles, cobbles, and boulders were placed on the ground to add nutrients, trap moisture, protect plants from wind, and stabilize temperature [28,41,42,67,69,86,87]. Apart from Poike and Rano Kau, mulch gardens are found across the island [28]. Cultivation also took place within small, circular-walled garden enclosures (*manavai*) that are thought to have been used primarily for taller cultigens such as bananas or sugarcane [67,68]. *Manavai* were likely of secondary importance to mulch gardens [19,88,89]. Terrestrial protein was available from domesticated chickens (*Gallus gallus*), rats (*Rattus exulans*), and potentially birds [90–92].

#### Marine resources

The relative importance of marine resources in the precontact Rapanui diet has been the subject of some debate. The first detailed ethnographies conducted in the early 20^th^ century (e.g., [82,93,94]) suggested that marine resources were a relatively unimportant dietary component at this time, though Métraux [94] suggested that fishing was more important in precontact times. These ethnographies documented a range of near-shore techniques used to target several eel and fish species, including netting, snaring, and hook and line fishing with stone, bone, and wooden fishhooks. These different kinds of fishhooks, netting needles, and stone net-sinkers are also found in archaeological contexts (e.g., [95,96]). Due to the paucity and small size of marine shell on Rapa Nui, shell fishhooks are unknown [95]. Near-shore foraging for invertebrates, such as octopus, crabs, lobsters, and urchins, was also practiced, though these were possibly of lower importance [95,97].

The available zooarchaeological data for Rapa Nui marine resource use are limited but also suggest the importance of near-shore fishing in the precontact times. Ayres [90,98] excavated several deposits on the north and south coasts and found that fish and shellfish remains comprised a relatively low percentage (< 30%) of the overall food remains and suggested a greater importance of near-shore fish taxa. Ayres [98] also found slight geographic differences in fish remains, with a higher proportion on the north coast, though these were balanced by a larger proportion of invertebrate remains on the south coast. However, a later analysis of additional remains from these sites found a much higher percentage (~85%) of fish remains and less geographic differences between the north and south coast [90]. Similarly, Rorrer [91] excavated two cave sites on the southwest coast which yielded abundant fish remains and small amounts of marine shell, with the assemblage being predominantly comprised of snapper, wrasse, and moray eel. Steadman et al.’s [92] excavations at Anakena beach also yielded an assemblage of fish and dolphin remains that were more or less evenly distributed through the deposit. While some have suggested that the presence of dolphin in these deposits implies that the Rapanui fished in the open ocean (e.g., [99,100]), it is more likely that dolphins were hunted in the shallows of Anakena bay as was done on other Polynesian islands [101]. Similar results from Anakena excavations are reported by Martinsson-Wallin and Crockford [102] and Hunt [38], though with a much higher abundance of fish remains. Apart from Steadman et al.’s [92] large assemblage of dolphin remains, these studies indicate the importance of nearshore taxa in precontact times. It should be noted that many of these studies used relatively large mesh screens (⅛ inch) during excavation (though Steadman et al. [92] subsampled material and screened with 1/16^th^ inch mesh for one square of their 1×4 m unit), thus potentially biasing our knowledge about marine resources against smaller fish and shell remains [103]. In sum, these studies all indicate the importance of nearshore taxa in precontact times.

Relative to terrestrial resources, marine resources were once thought to make only a small contribution to subsistence [104–106]. A recent reanalysis of the stable isotope evidence [107], however, indicates that marine resources composed at least 50% of dietary protein. In conjunction with the zooarchaeological evidence, these findings demonstrate that marine resources played a significant role in the Rapanui diet. Thus, while Rapa Nui’s marine resources are limited when compared to other Polynesian islands, they nonetheless comprised an important part of the precontact subsistence system. In addition, recent surveys of Rapa Nui’s marine ecosystem indicate that overall fish biomass is relatively high (e.g., [44]). Rapa Nui’s relatively marginal marine environment, however, restricted opportunities for prehistoric populations to intensify reef foraging (especially for shellfish), a practice important elsewhere in Polynesia (e.g., [108,109]).

#### Freshwater

Freshwater is a limited resource of critical importance that is infrequently discussed for Rapa Nui. As noted above, the island receives a moderate amount of rainfall but also experiences frequent droughts and, due to soil and bedrock permeability, has no permanent streams. The highly permeable bedrock allows water to rapidly transfer to the island’s unconfined aquifers, and the water table is generally only a few MASL near the coast [43]. Herrera and Custodio [43] have suggested that groundwater may be perched on less permeable geologic features inland, but except where a few springs occur, the island’s geology makes inland groundwater inaccessible without the aid of modern drilling equipment. While there are a few isolated instances of landforms that give the impression of once being fluvial ravines [96,110], these are likely volcanic features such as collapsed lava tubes [111].

Freshwater is also available in the island’s many lava tubes, found mainly on the western end of the island, where groundwater and rainwater can collect [43,82,96,112]. The only large perennial bodies of freshwater are lakes and springs resting atop impermeable portions of volcanic cores in Rano Kau, Rano Raraku, and Rano Aroi. However, there is a curious lack of evidence for these lakes being primary water sources in either pre- or post-contact times (e.g., [82,94];cf. [113,114]), likely due to their inaccessibility or distance from the majority of habitation areas. The highly permeable substrate and absence of perched aquifers led to the primary challenges faced by the Rapanui in procuring freshwater.

Early European visitors were quick to note the scarcity and brackish quality of freshwater on Rapa Nui, and their reports also provide key insights into the primary sources of freshwater. At first European contact in 1722, Bouman, a captain in Roggeveen’s Dutch expedition, noted that the Rapanui had “calabashes [i.e., gourds, *Lagenaria* sp.] in which they kept water which I tasted and found to be quite brackish.” [115]. Later visitors also describe the use of gourds for water storage and transport (e.g., [116,117]). Due to its ability to retain considerable moisture, sugarcane was also possibly used as a water source [116,118]. However, the historical accounts of sugarcane use are contradicted by the relatively low abundance of their phytoliths in human dental calculus [119]. Most observations point to the importance of freshwater from coastal areas. Cook, for example, noted that the islanders drank coastal water, commenting that the water given to them by the Rapanui was “brackish and stinking” which was only “rendered acceptable by the extremity of their thirst” and later saying that they were even given “real salt water” and that the Rapanui “drank pretty plentifully” from the sea [120]. From these descriptions, it is likely that Cook experienced the Rapanui using coastal groundwater discharge (CGD) whereby groundwater seeps up in many locations along the island’s coastline. There are several additional European accounts of the Rapanui drinking seawater (e.g., [116,118,121]), though, as pointed out by Routledge [82], these were almost certainly observations of the use of CGD as freshwater sources. These European accounts indicate that CGD was an important freshwater source for the Rapanui [29].

Archaeologically, evidence for freshwater management occurs mostly in the form of features known as *taheta* and *puna*. *Puna*, sometimes referred to as ‘wells,’ are stone paved and sometimes walled features occurring along the coast that served to trap CGD [93,94]. Métraux [94] recognized the important function of *puna*, noting these features “impounded rain water and perhaps some fresh water springs”, and that the “ruins of ancient settlements are always thick around water holes” [94]. In his early ethnographic and archaeological surveys, Englert [93] also noted the co-occurrence of water sources with both *ahu* and settlements.

In addition to the use of *puna*, the Rapanui also made freshwater features known as *taheta*. *Taheta* are small (i.e., <1 m wide) and shallow rainwater basins carved into basalt bedrock, which, being dependent on rainfall, provided opportunistic and temporary sources of freshwater [111]. These features can be found scattered throughout the island, though they appear to be more abundant in inland areas and on the northwest coast [59,119,122]. Freshwater diatoms extracted from the dental calculus of precontact human remains [119] suggest that populations relied on features like *taheta* that would have been habitat for phytoplankton. Many of the diatoms identified in the skeletal remains prefer brackish water [123], indicating the islanders also used other kinds of standing pools located at or near the coast, such as brackish water at locations of CGD or *puna*. In addition to *taheta* and *puna*, Vogt and colleagues [124,125] have identified a unique water basin and possible dam feature at the inland site of Ava Ranga Uka A Toroke Hau. In sum, while Rapa Nui lacks many obvious sources of freshwater, the geology of the island, the distribution of archaeological material, and ethnohistorical accounts suggest a heavy reliance on water from coastal areas, in particular CGD, which was at times impounded through the use of *puna*.

Below, we present a series of spatially-explicit models designed to assess the degree to which image-*ahu* spatial locations are explained by the presence of rock mulch gardens, marine resource locations, and/or freshwater sources to test competing hypotheses about whether monument construction on Rapa Nui is related to subsistence resource constraints.

## Materials and methods

### Archaeological and environmental data

**Fig 2:**
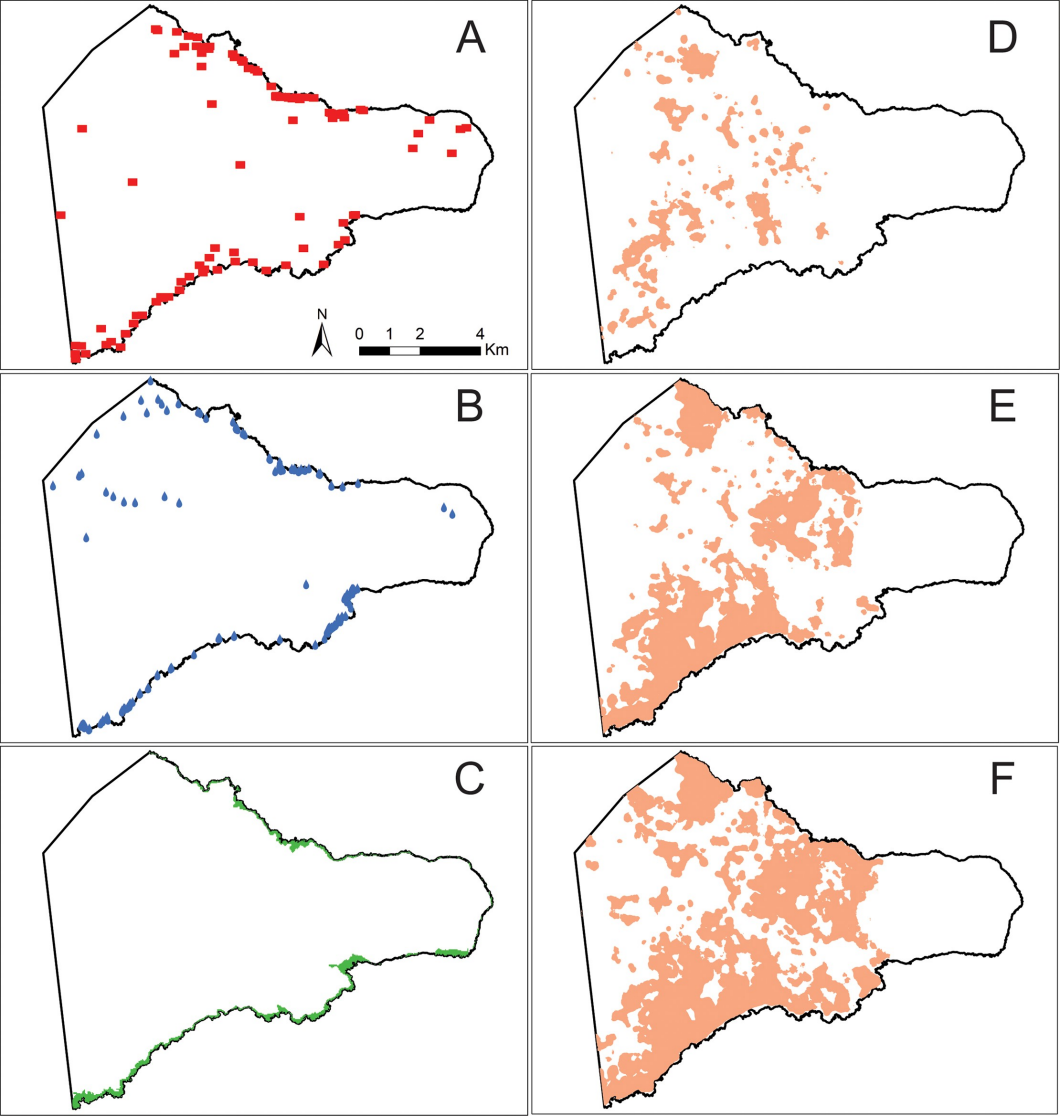
Archaeological and environmental data used in our analyses. (A) locations of image-*ahu*, (B) locations of freshwater sources, (C) marine resource locations, (D) minimal rock mulch classification, (E) medial rock mulch classification, (F) maximal rock mulch classification.

To compare the spatial distribution of *ahu* to subsistence resource locations requires comprehensive spatial coverage of these variables, and we therefore restrict the current analysis to an eastern region of Rapa Nui (Fig 2) where we have data on monuments, agricultural plots, marine resource locations, and freshwater sources. Research permits for our study were provided by Comunidad Indígena Polinésica Ma‘u Henua, the Easter Island Development Commission (CODEIPA), the Chilean Consejo de Monumentos Nacionales, and National Forestry Corporation (CONAF). The 93 image-*ahu* and their locations derive from the comprehensive *ahu* survey conducted by Martisson-Wallin [10]. We determined *ahu* locations by georeferencing Martinsson-Wallin’s [10] maps and subsequently correcting their locations during field surveys using a Trimble Geo 7x GPS unit and with Google Earth imagery (Fig 2A).

Our analyses of agricultural resources focus on the relationship between image-*ahu* and rock mulch gardens, rather than other features like *manavai* or agricultural productivity models (e.g., [59,126]), as these features represent known locations of the most intensified agricultural production. The locations of rock mulch gardens derive from the results of Ladefoged et al. [28], who produced remote-sensing-based documentation of the distribution of mulch gardens across the island. Ladefoged et al. [28] produced three products from their analyses: minimal, medial, and maximal mulch classifications. We analyzed each of these three mulch classifications. To facilitate the distance-based analyses outlined below, we transformed the rock mulch dataset. First, we converted the data into a binary raster with areas of rock mulch = 1 and non-mulch areas = 0. Next, we created a mulch density estimate by calculating the mean occurrence of rock mulch within a circular neighborhood of 100 m around each cell, and this result was reclassified into a new binary raster with 1 = the upper 90% (Fig 2D–2F). This process filtered out potential noise in Ladefoged et al.’s [28] rock mulch model that would come from small areas being incorrectly classified as mulch or small isolated areas unlikely to be important resource locations, based on previous hypotheses that only the largest intensified field systems were associated with construction of image-*ahu* (e.g., [67–69]). We then created a distance map for rock mulch, i.e., a raster layer where each cell value is equal to its Euclidean distance from rock mulch gardens.

As discussed above, Rapa Nui lacks large coral reefs and has a relatively homogenous rocky marine environment. Given these characteristics of the island’s marine ecology and archaeological evidence for the importance of near-shore taxa, the most suitable locations for marine resource procurement would have simply been areas with easy coastal access. Using NASA’s Shuttle Radar Topography Mission (SRTM) 30 m digital elevation model (DEM), we defined marine resource locations as areas <10 MASL (Fig 2C). This choice of 10 MASL is somewhat arbitrary, but captures the locations of low-elevation embayments, which would have provided the best access points for near-shore fishing and other forms of marine foraging [18,127]. To explore the sensitivity of our modeling to this <10 MASL cutoff, we also perform these analyses using a <5 MASL threshold (S8 File). As for the rock mulch data, we created a Euclidean distance map with cells equal to distance from marine resource locations.

Our freshwater data derive from previous studies (e.g., [43,123,128]) and our ongoing pedestrian and geochemical surveys [29,30] designed to locate precontact freshwater sources, such as lakes, springs, ponds, caves with seeping groundwater, *puna*, and coastal seeps (Fig 2B). We do not consider *taheta* in our analysis for the same reason we filtered out smaller areas of rock mulch. Specifically, *taheta* would have provided only small and temporary sources of freshwater and thus would have been unlikely sources of competition [29]. Complete survey data on *taheta* are also presently unavailable. Coastal seeps, or coastal groundwater discharge (CGD), locations were identified using EXTECH EC170 salinity meters, which measure the concentration of dissolved ions in coastal water expressed in parts per thousand (ppt). Salinity measurements were taken every 10 m along the coastline within our survey area (Fig 2B) and within one hour of low-tide when CGD is at its maximum. Coastal seeps were defined as locations where the salinity of coastal water was substantially less than the average salinity of seawater (ca. 35 ppt), in this case 28 ppt or less. This value represents a 20% reduction in salinity and therefore signals areas of substantial CGD that could be used as freshwater sources (further details in [29]). In limited locations we also identified CGD using a Solonist Levelogger conductivity meter based on differences in conductivity between freshwater and seawater [129]. Rapa Nui seawater has a mean conductivity value of 55 millisiemens (mS), so any location with a conductivity <50 mS was classified as a high concentration of CGD [130]. While we have not yet resolved the question of potential temporal variability in discharge rates, given Rapa Nui’s hydrogeology, the spatial locations of these water sources have likely remained stable over the time period of human occupation [29,43]. These freshwater data were transformed into a Euclidean distance map where cell values equal distance from freshwater sources.

### Point process modeling

Two fundamental concepts in spatial analysis are the first- and second-order properties of point patterns [25,131]. The first-order property of a point pattern is its intensity, defined as the number of points per unit area of the study region. The intensity is usually described as being homogeneous (i.e., expected number of points the same across the study area) or inhomogeneous (i.e., spatially varying intensity) [24]. The second-order property is the interaction among points, such as clustering (resulting from an attraction process) or dispersion (resulting from some kind of repulsion/spacing). In general, a major goal of point-pattern analysis is to account for what, if any, independent variables explain the intensity of the point pattern and whether aspects of the spatial pattern are accounted for by clustering/dispersion among points. These two properties are analytically important to distinguish, as aspects of first-order intensity may be conflated for second-order interaction, such as a set of points appearing clustered simply due to their tendency to be in one part of a study area. On Rapa Nui, for example, one might assume that the tendency for *ahu* to be dispersed along the coastline is related to settlement spacing (second-order property), though this distribution may be sufficiently accounted for by a dependent relationship with coastal resources (inhomogeneous first-order intensity). It is also possible that *ahu* spatial patterns are explained by both first- and second-order properties. Below, we apply a series of formal techniques for modeling these two properties in an effort to better understand which properties and variables best explain the spatially varying intensity of image-*ahu*.

To test whether geographic patterns of *ahu* construction are explained by resource availability, we evaluate the following hypotheses related to the spatial dependence of *ahu* on distance from subsistence resources locations: (1) image-*ahu* have a homogeneous or inhomogeneous random spatial distribution (null hypothesis), (2) image-*ahu* have an inhomogeneous spatial distribution which is simply explained by distance from the coastline; image-*ahu* have an inhomogeneous spatial distribution that is best explained by (3) distance from rock mulch gardens; (4) distance from marine resource locations; (5) distance from freshwater sources; or (6) the spatial distribution of image-*ahu* is explained by some combination of these variables.

We assess hypothesis 1 using homogeneous and inhomogeneous forms of Besag’s L-function [132,133]. The homogeneous L-function simply tests for deviations from complete spatial randomness (CSR) in the form of clustering or dispersion in a point-pattern at different spatial scales, whereas the inhomogeneous L-function tests for clustering or dispersion relative to the inhomogeneous intensity of the underlying point-pattern. For example, *ahu* may appear clustered simply because they are predominantly coastal in their distribution, though once we account for this spatial inhomogeneity they may be neither clustered nor dispersed. We assess the statistical significance of these tests using Monte Carlo simulation envelopes of CSR. Areas of the empirical function falling outside this envelope indicate significant departures from CSR, with areas above the envelope indicating clustering and below the envelope indicating dispersion. Here, we use 39 simulations of CSR, which is equivalent to testing at the *p* = 0.05 level.

To test hypotheses 2 through 6, we first explore potential relationships between *ahu* and distance from subsistence resource locations using spatial Kolmogorov-Smirnov (SKS) tests [134]. The SKS test works by comparing the spatial empirical cumulative distribution function (CDF) to the expected CDF under the null hypothesis of CSR and thus provides an indication of whether the spatial distribution of a point-pattern is non-randomly patterned according to an underlying spatial covariate. We perform this SKS test for *ahu* spatial relationships with distance from marine resource locations, freshwater sources, and Ladefoged et al.’s [28] three rock mulch classifications. The alternative hypothesis is that the CDF for *ahu* lies above that expected under CSR, i.e., that *ahu* are closer to these resource locations than is expected for a random spatial pattern. The SKS test, however, is merely an exploratory tool to help guide the choice of spatial covariates to use in more formal models, and here those spatial covariates for which *ahu* are non-randomly constructed near are further evaluated using point-process modeling.

Point-process models (PPM) are a wide class of spatially explicit models that facilitate formal analysis of the relationship between point-patterns and a range of spatial covariates [24]. PPM works by fitting a spatial intensity function to the intensity of an empirical point pattern and finding the values of the predictor variables (i.e., parameters) that best fit the data [24]. The technique is similar to geographically weighted regression or maximum entropy modeling but has a number of strengths [24], such as its ability to simultaneously model both first-order (i.e., homogeneity/inhomogeneity) and second-order (i.e., clustering/dispersion) properties in the underlying point-pattern and how these properties may be dependent upon a set of underlying spatial covariates [25,26]. PPM is therefore well-suited to the objectives of this study.

Rather than simply evaluate the likelihood of different models or test for significant effects of different spatial covariates, PPMs allow for the use of formal model-selection tools based on information criteria. Tools like the Akaike Information Criterion (AIC; [135]) or Bayesian Information Criterion (BIC; [136]) allow for the formal comparison of competing potential models about the formation of archaeological patterns. These tools are based on a principle of parsimony, which penalizes models for additional parameters, so the model chosen as ‘best’ is the one that explains the most variability in the underlying data in the simplest way. This parsimony criterion is beneficial, because more complex models often will have higher likelihoods simply because of additional parameters, though they may be overfit [26]. The use of information criteria therefore allows us to evaluate the tradeoff between model complexity and likelihood in selecting the best model. One convention is to choose the model which has the smallest change in information criterion score (e.g., ΔAIC or ΔBIC) and the highest weight, which provides a measure of the relative strength of different candidate models [26,137].

To accomplish this task, we built a series of inhomogeneous Poisson PPMs that model the log-linear relationship between an empirical point pattern and different spatial covariates, in this case how the spatial trends in *ahu* construction are predicted by subsistence resource locations. Our initial model simply considers the inhomogeneous relationship between *ahu* locations and their distance from the coastline, i.e., based on the possibility that *ahu* are not related to subsistence resources but simply occur in coastal areas (hypothesis 2). We then built additional models which consider the spatial dependence of *ahu* on distance from different combinations of subsistence resource locations (hypotheses 3–6). Following the suggestion of Kuha [138], we then use both ΔAIC and ΔBIC to formally compare these models, for when used in tandem these two information criteria can be powerful tools for selecting the best-fitting model.

Once the best-fitting model was selected, we evaluated the fit between the model and the data using a number of techniques. First, we used the residual L-function, which compares whether the L-functions of simulated realizations from the best-fitting model are statistically indistinguishable from the L-function of the empirical point-pattern [24]. Regions of the empirical function falling outside the envelope of the L-function for the model indicate a poor fit between the model and the data. For example, if the L-function of the empirical pattern falls above the envelope for the model, then the empirical pattern is likely more clustered than was accounted for in the model. We also assessed the fit between the L-function of a PPM and empirical point-pattern by implementing the maximum absolute deviation (MAD) and Diggle-Cressie-Loosmore-Ford (DCLF) tests using 39 Monte Carlo simulated realizations of the model [139]. For these latter two tests, high p-values indicate good fit between the model and data while low p-values suggest significant deviations between them. In the event of poor fit, the models can be re-parameterized to include second-order properties such as clustering or dispersion. We also present visualizations of simulated realizations of the best-fitting PPM using the Metropolis-Hasting algorithm [140]. We performed our analyses with R [141], using the spatstat package for PPM [24] and the MuMIn package for multi-model selection [142]. All data (S1–S6 Files) and R code (S7 and S8 Files) necessary for running these analyses are available in supplementary files.

## Results

**Fig 3:**
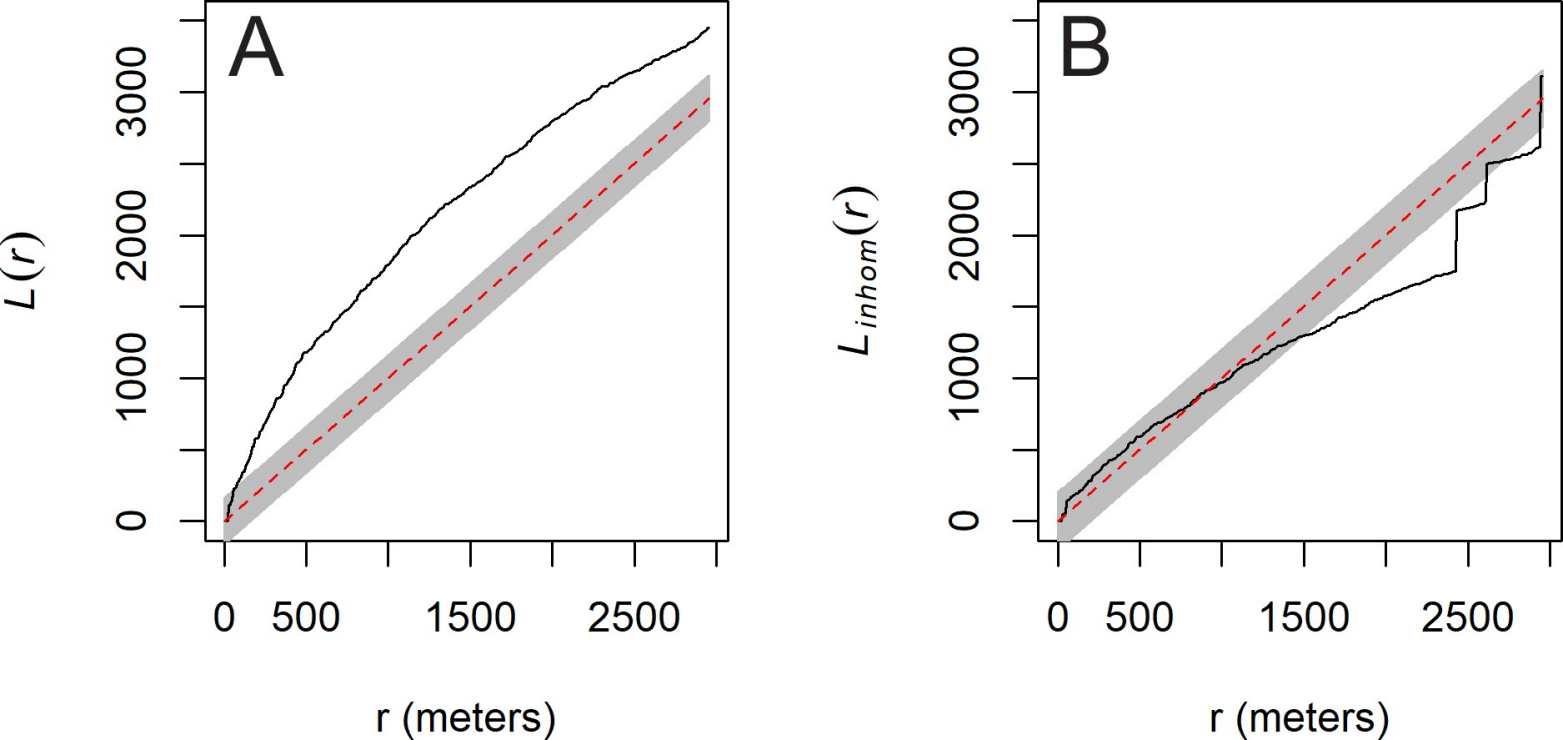
Test of hypothesis 1. (A): L-function of *ahu* compared to 39 simulated realizations of CSR; (B): inhomogeneous L-function of *ahu* compared to 39 simulated realizations of CSR. Y-axes are the values of the L-functions at separation distances (r) in meters (x-axes). Results indicate that image-*ahu* have an inhomogeneous intensity lacking second-order properties, though there is some evidence for dispersion at distances >1500 m. Black lines are the empirical L-functions, red dashed lines are the theoretical expectations under the null model, and the grey-shaded region is the envelope of 39 Monte Carlo simulations of the null model.

Fig 3 shows the results of the homogeneous and inhomogeneous L-function, which tests for deviations from CSR in the *ahu* point pattern. In the figure, the black line is the empirical L-function for *ahu*, the red line is the expectation under CSR, and the grey regions are the *p* = 0.05 significance envelopes. Fig 3A shows that when compared to CSR *ahu* appear highly clustered at nearly all spatial scales, though when accounting for the spatial inhomogeneity of *ahu* (Fig 3B) they are neither clustered nor dispersed, except for dispersion at distances greater than ca. 1500 m. As expected, these results indicate that *ahu* have an inhomogeneous spatial distribution but little evidence for clustering or dispersion at distances less than 1500 m. Next, we model whether this inhomogeneous spatial distribution is explained by one or more environmental variables.

**Fig 4:**
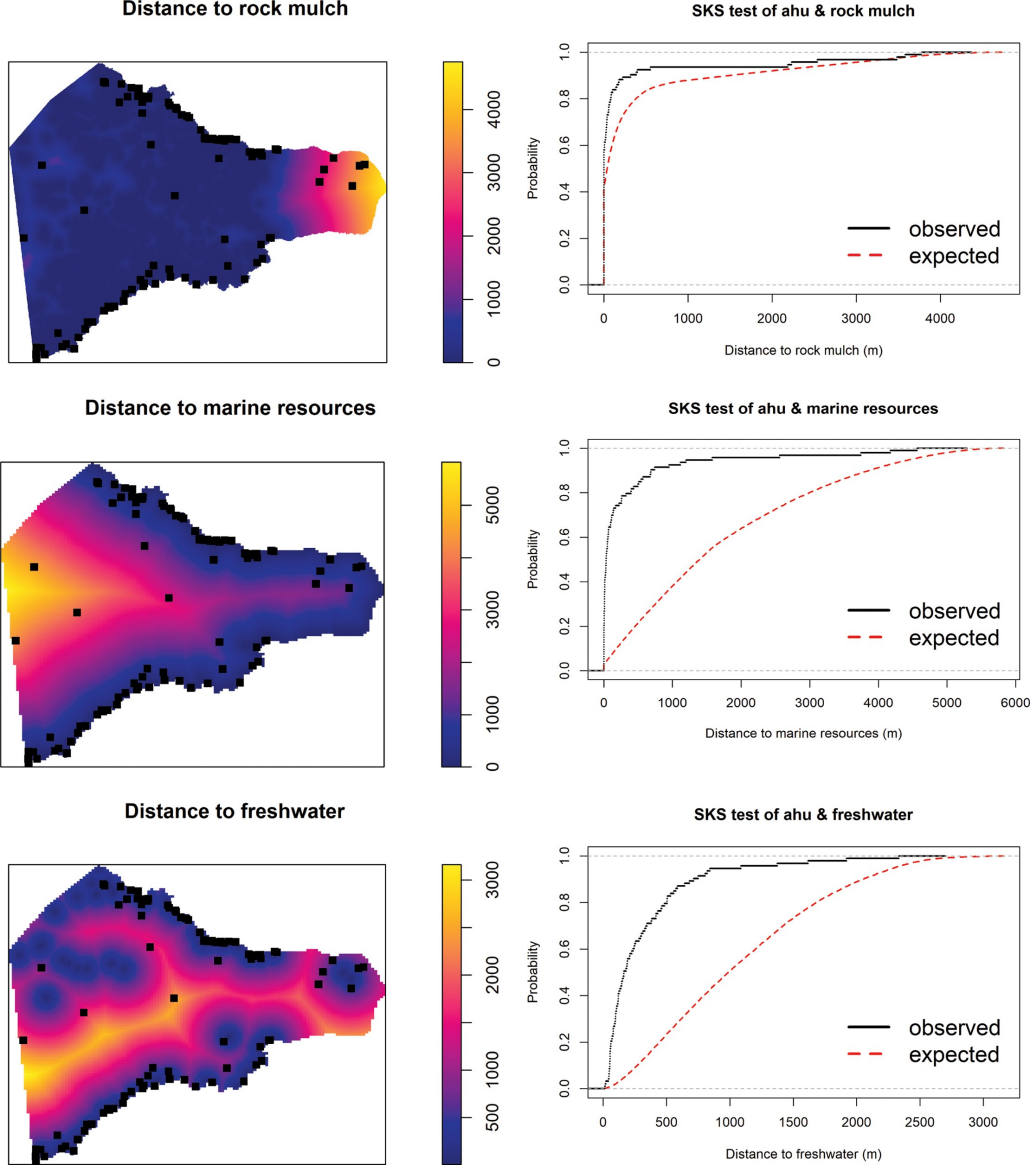
Spatial Kolmogorov-Smirnov (SKS) tests. SKS tests for the relationship between image-*ahu* (black squares) and distance (m) from subsistence resource locations (choropleth maps). Observed distribution (black lines) is compared to the expected distribution under CSR (dashed red lines) with the alternative hypothesis being that *ahu* are nearer to these resources than random. Results suggest *ahu* are significantly clustered near freshwater sources (D^+^ = 0.59, p<0.0001), marine resource locations (D^+^ = 0.65, p<0.0001), and the maximal rock mulch garden classification (D^+^ = 0.24, p<0.0001). Results for minimal and medial mulch classifications can be found in S8 File.

Fig 4 shows the results of the SKS tests on the relationship between *ahu* and marine resource locations, freshwater sources, and rock mulch gardens. The results indicate that *ahu* are not significantly closer to the minimal rock mulch classification than expected under CSR (D^+^ = 0.083, p = 0.26); however, there is a significant spatial association for both the medial (D^+^ = 0.20, p = 0.0004) and maximal classifications (D^+^ = 0.24, p < 0.0001) from Ladefoged et al. [28]. *Ahu* are significantly closer to both marine resource locations (D^+^ = 0.65, p < 0.0001) and freshwater sources (D^+^ = 0.59, p-value < 0.0001) than expected under CSR. Based on these findings we then formally explored the relationship between *ahu* and marine resource locations, freshwater sources, the maximal rock mulch classification, and the coastline using PPM and multi-model selection. We also applied the same PPM procedure using the medial rock mulch classification and a <5 MASL threshold for marine resource locations and obtained similar results (see S8 File).

Table 1 shows the different candidate models with their ΔAIC, ΔBIC, and weights. Both model selection tools indicate that *ahu* spatial patterns are poorly explained by just distance from the coastline, suggesting that some additional spatial covariate is needed to explain the distribution of *ahu*. Both AIC and BIC indicate that *ahu* locations are best explained by an additive model with the combined effects of distance from the coastline and distance from freshwater sources (model 5) with a ΔBIC of 0 and a BIC weight of 0.675 and a ΔAIC of 0 and an AIC weight of 0.35.

**Table 1:** Point-process model selection for the relationship between *ahu* and subsistence resources. Smaller change in information criteria score (ΔBIC and ΔAIC) and higher weight indicate best-fitting model.

*Model*	*Covariates*	*df*	*ΔBIC*	*ΔAIC*	*BIC weight*	*AIC weight*
1	coastline	2	49.61	52.14	0	0
2	freshwater	2	54.62	57.16	0	0
3	marine resources	2	61.36	63.89	0	0
4	rock mulch	2	231.19	233.72	0	0
**5**	**coastline + freshwater**	**3**	**0**	**0**	**0.675**	**0.350**
6	coastline + marine resources	3	51.91	51.91	0	0
7	coastline + rock mulch	3	35.28	35.28	0	0
8	freshwater + marine resources	3	5.51	5.51	0.043	0.022
9	freshwater + rock mulch	3	58.94	58.94	0	0
10	marine resources + rock mulch	3	50.51	50.51	0	0
11	freshwater + marine resources + rock mulch	4	10.02	7.49	0.005	0.008
12	Coastline + freshwater + marine resources	4	2.66	0.13	0.178	0.328
13	Coastline + freshwater + rock mulch	4	4.36	1.83	0.076	0.140
14	Coastline + marine resources + rock mulch	4	34.88	32.34	0	0
15	Coastline + freshwater + marine resources + rock mulch	5	6.75	1.68	0.023	0.151

**Fig 5:**
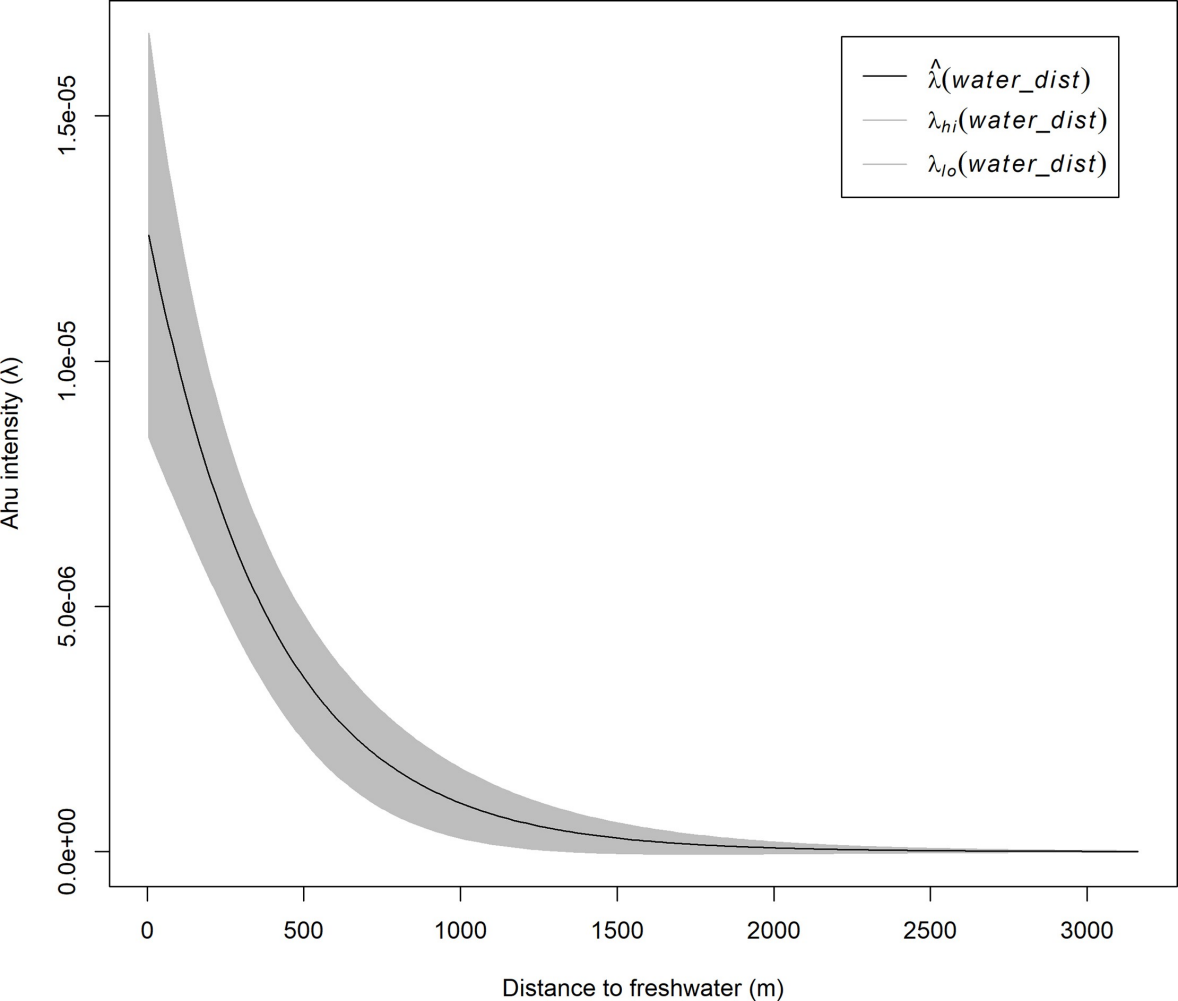
Effect of distance from freshwater sources on the spatial intensity of image-*ahu*. *Ahu* spatial intensity declines with distance from freshwater sources. Grey-shaded region represents the 95% confidence interval.

Table 2 shows the covariate estimates, standard errors, 95% confidence intervals, and Z values for the best fitting model 5. Negative values of the covariate estimates indicate that *ahu* intensity decreases with distance from the coast and freshwater sources, i.e., the inhomogeneous intensity of image-*ahu* is greatest near these resources. Fig 5 graphically displays the inverse relationship between the effect of distance from water sources on the intensity of *ahu*.

**Table 2:** Covariates for best-fitting model 5. Negative covariate estimates indicate that *ahu* intensity decreases with distance from the coast and freshwater sources.

Covariate	Estimate	Standard error	95% Confidence interval, low	95% Confidence interval, high	Z-test	Z-value
*(Intercept)*	-11.2	0.16	-11.6	-0.1	<0.0001	-70.06
*Distance from coastline*	-0.001	0.0003	-0.002	-0.0007	<0.0001	-4.7
*Distance from water*	-0.003	0.0004	-0.003	-0.002	<0.0001	-5.9

**Fig 6:**
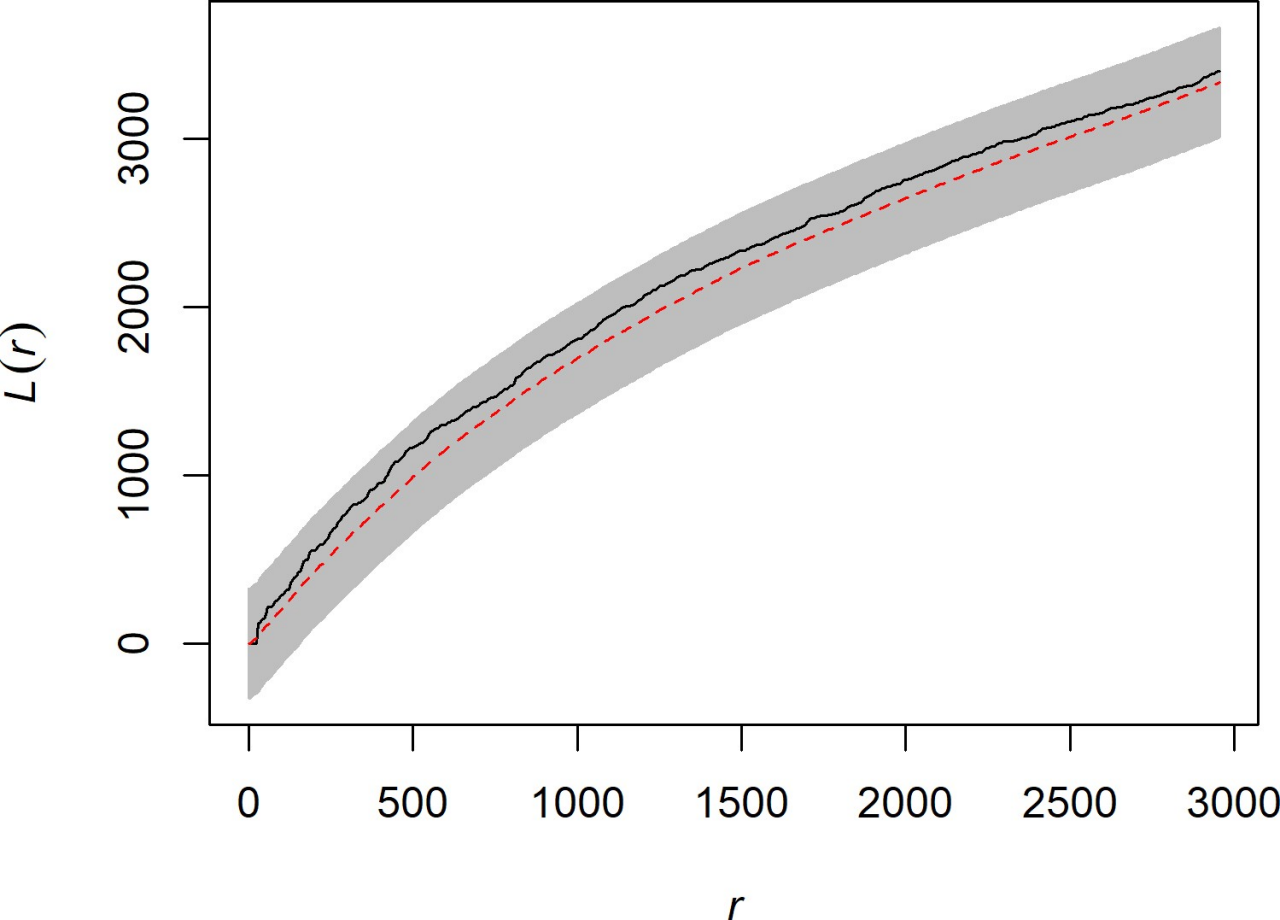
Residual L-function for best-fitting model. Red dashed line is the theoretical L-function of the model, grey shaded region represents the upper and lower bounds of 39 Monte Carlo simulated realizations of the model (*p* = 0.05), and the black line is the L-function for *ahu*. Y-axis is the value of the L-function at distances (r) in meters (x-axis) Results indicate no significant deviation between model 5 and the data.

Fig 6 shows the result of the residual L-function test, which serves as a form of model validation. The results indicate no significant deviations between model 5 and the data. To further validate the model, we also used a MAD and DLCF test [139]. The results of these tests indicate that model 5 is a good fit to the data (MAD = 171.91, p = 0.22; U = 41528000, p = 0.18). This finding indicates that the inhomogeneous spatial intensity of image-*ahu* is sufficiently explained by the model 5 covariates and that no second-order interaction parameters (e.g., clustering/dispersion) are warranted, thus pointing to the significance of freshwater sources and their association with *ahu* locations.

**Fig 7:**
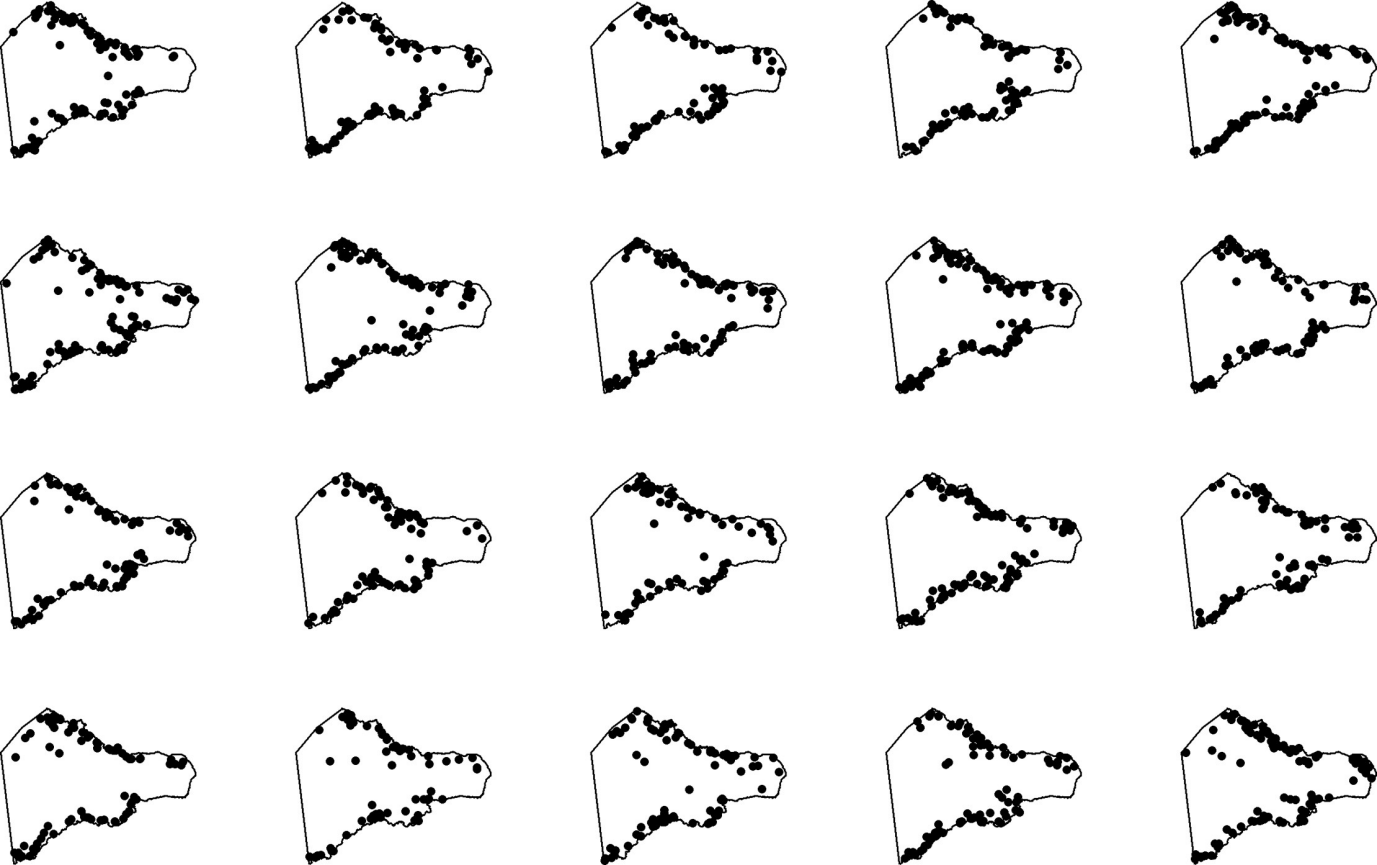
Simulated realizations of the best-fitting model. 20 simulated realizations of the best-fitting model 5 incorporating distance from the coastline and distance from freshwater sources.

Our final step in model validation is to visually compare simulated realizations of the best-fitting model 5 against the empirical point pattern using the Metropolis-Hasting algorithm [140]. Fig 7 shows 20 simulated realizations of the best-fitting model. The results show that the model produces point patterns that are primarily coastal in distribution but with a few scattered points inland, similar to the empirical distribution of image-*ahu* (Fig 2A).

## Discussion

Our results indicate that the image-*ahu* point-pattern exhibits neither clustered nor dispersed second-order properties but that their inhomogeneous coastal distribution is sufficiently explained by a dependent relationship with some subsistence resources, though not others. Our findings do not support the claims that *ahu* are related to competition over or monitoring of agricultural resources, at least in the sense that the distribution of image-*ahu* are not explained by the locations of rock mulch gardens, which were the source of intensified sweet potato production (e.g., [67–70]). While the construction of Rapa Nui’s monuments may be coeval with the establishment of rock mulch field systems, image-*ahu* do not appear to be built in locations to mark control or territoriality over these resources. This result is supported by other evidence indicating a lack of control over other resources, in particular fine-grained basalt, *moai* tuff, and red scoria (e.g., [78,79];cf. [77]).

While our exploratory SKS analyses suggest a strong and significant spatial association between *ahu* and marine resource locations, our PPM and multi-model selection suggests this variable is not particularly meaningful. It is likely that marine resource locations appear correlated with image-*ahu* due to their geographic proximity to the more meaningful explanatory variables (coastline, freshwater). That is, because marine resource and freshwater locations tend to occur in similar locations, significance tests show that *ahu* are significantly related to both. It is in this kind of situation where multi-model selection is useful. Based simply on summary statistics and significance tests, image-*ahu* appear to be located near prime marine resource access points; however, marine resource locations are not necessary to explain the spatial patterns of *ahu*. These results highlight the strengths of formal model selection over the more common method of significance testing in archaeological analyses (e.g., [26,143,144]). Significantly, our results offer little support to recent claims that *ahu* were preferentially built at locations that mark control over marine resource locations (e.g., [18]). As emerging lines of evidence indicate that marine resources were likely not as limited in the past as once thought (e.g., [107]), they may not have been the focus of inter-community resource competition.

Our multi-model selection indicates that, in addition to their primarily coastal distribution, image-*ahu* spatial patterns are most parsimoniously explained by an inhomogeneous Poisson PPM that models the spatial trend using distance from freshwater sources. Our multiple model validation procedures show that, when accounting for these covariates, no second-order properties are needed to explain the spatial intensity of image-*ahu*. Overall, simulated realizations of the model (Fig 7) produce patterns that are remarkably similar to the empirical *ahu* pattern, with most points occurring near the coast but with a few scattered inland. This suggests that our model 5, which incorporates distance from the coastline and freshwater sources, captures the underlying spatial patterns of *ahu* well.

This result is significant, as it likely explains the previously unresolved issue of why Rapa Nui’s monuments occur primarily along the coast–one the most abundant sources of freshwater, coastal seeps, occurs primarily in coastal locations. The fact that our model 5 is a much better fit than the simpler model 1, which only includes distance from the coastline, offers compelling support for the claim that *ahu* are related to freshwater locations.

While one might argue that these results are unsurprising given that human settlements tend to be associated with freshwater sources, this would not account for important characteristics of the settlement pattern on Rapa Nui, which, like elsewhere in Polynesia (e.g., [14,145]), is characterized by ritual architecture being spatially distinct from domestic settlement clusters. Specifically, areas of domestic activity are slightly disassociated from *ahu* and tend to occur 100–200 m inland [19,59,60]. While these separation distances between domestic and ritual activity are not great in an absolute sense, what is notable is here is the non-random relative patterning in the topology of domestic features and monument locations. For example, Morrison [59] found that clustered sets of co-occurring domestic activity areas were spatially segregated from the locations of *ahu*. However, at larger spatial scales, groups of these redundant sets of domestic features, while located further inland, are generally clustered around one or more *ahu*. In other words, while *ahu* were indeed the focal points for the settlement pattern, they are spatially offset from them, and directly adjacent to freshwater sources. These patterns suggest a link between freshwater locations and the factors underlying the emergence of ritual monument construction on the Rapa Nui landscape.

One interpretation of these results is that *ahu* were preferentially built near freshwater sources to demarcate community access/control over these resources. This interpretation draws on the logic of costly signaling theory, whereby Rapa Nui’s monuments are hypothesized to serve as conspicuous displays of community access/control over the island’s limited subsistence resources. Recently, such a costly signaling model for *ahu* has been proposed (e.g., [8,12,16,17]), which predicts that, if Rapa Nui’s monuments did serve a costly signaling function, then there should be a spatial association between them and the underlying quality they are potentially signaling, such as the limited and vitally important, freshwater resources. These predictions are quantitatively supported by our present results. Such an interpretation is not unique. McCoy [19], who conducted the first large-scale settlement pattern analysis on Rapa Nui, suggested that warfare in precontact times would have likely been over freshwater. It is interesting to note that McCoy [19] also suggested a signaling function for *ahu* and argued that, “[e]laboration of…ahu…would, from present knowledge, provide a rough index of success in competition between lineages…based ultimately on the free time that could be allotted to such non-vital activities.” Several emerging and independent lines of evidence show that there is little empirical support for violent warfare, including little evidence for the production of lethal weapons [75,146], limited instances of lethal skeletal trauma [147], and a lack of fortifications [17,148–150]. Given this lack of evidence for warfare, it is possible that inter-community competition took the form of territorial displays, or costly signals, through the construction of monumental architecture directly adjacent to the island’s limited freshwater locations. However, additional formal analyses to specifically test these ideas are needed to evaluate this scenario.

One limitation of our analysis is the lack of explicit spatio-*temporal* analyses of *ahu*. Unfortunately, such a comprehensive temporal analysis is not possible at this time given the overall lack of secure and high precision radiocarbon dates for Rapa Nui [9,46]. We can say, however, that the construction of *ahu* likely began shortly following colonization and within ca. 500 years they numbered in the many hundreds. Temporal changes in the island’s environment and resources have been the subject of intensive research and prolonged debate. Regarding changes in freshwater availability, several researchers have suggested that the loss of the island’s palm forest severely degraded the amount of available surface freshwater (e.g., [92,110,114,124,151]); however, little in the way of hydrologic rationale nor data have been presented to suggest this would be the case. Lake-core sediment data suggest a possible prolonged period of drought from the 16^th^ to 18^th^ centuries (e.g., [33,152]) that would have reduced available freshwater from precipitation. These potential climatic and landscape changes would have only increased the vital importance of freshwater coastal seeps. Given the island’s hydrogeology, the locations of these freshwater sources (and also marine resource locations) have likely remained stable over time, and as many have argued, the growth of the island’s lithic mulch field systems is coeval with the construction of monumental architecture (e.g., [67,69,70]). Therefore, we see our spatial analyses as an investigation of the processes that led to the formation of Rapa Nui’s monumental landscape–a spatial pattern which appears best explained by the location of freshwater sources. However, an extension of our analysis to the western portion of Rapa Nui, once freshwater data from that region become available, will be necessary to more fully explore these spatial patterns of monument construction.

## Conclusions

The contrast between Rapa Nui’s marginal environment and the degree of investment in monumental architecture has puzzled researchers since first European contact. The long held orthodox view assumed that the island must have supported a larger and more complex society under more prosperous environmental conditions that then ‘collapsed’ following a self-imposed ‘ecocide’ [100]. In recent years, nearly every major component of this narrative has been shown to lack empirical sufficiency (e.g., [12,46,78,150]). A key finding is that the construction and transport of the island’s *moai* and *pukao* (red scoria ‘hats’) required neither large numbers of individuals nor trees [54,57]. The implications of this are far-reaching, particularly in that they question the common assumptions that monument construction necessarily involved complex social organization and labor management or that it necessarily led to environmental degradation (e.g., deforestation, erosion, etc.). Major unresolved issues, however, concern the labor invested and choices made in constructing *ahu*, in particular why *ahu* (and the *moai* and *pukao* upon them) were built where they were and how monument construction might relate to territorial signaling of control over subsistence resource availability. Here, we have presented a series of formal models which indicate that if Rapa Nui’s monuments did indeed serve a territorial display function (in addition to their well-known ritual roles), then their patterns are best explained by the availability of the island’s limited freshwater.

## Supporting information

S1 File Survey area. Zip file containing polygon shapefile for our study area on the eastern portion of the island.
(ZIP)

S2 File Ahu. Zip file containing point shapefile of image-*ahu* within the study area.
(ZIP)

S3 File Rock mulch. Zip file containing Euclidean distance maps for the minimal, medial, and maximal rock mulch classifications.
(ZIP)

S4 File Marine resource locations. Zip file containing polygon shapefiles for marine resource locations.
(ZIP)

S5 File Freshwater sources. Zip file containing point shapefile of the locations of freshwater sources within the study area.
(ZIP)

S6 File Coastline. Zip file containing Euclidean distance map for distance from the coastline.
(ZIP)

S7 File Rmarkdown. Rmarkdown file necessary to create figures and execute all statistical analyses.
(RMD)

S8 File PDF of Rmarkdown. PDF file showing the output of running the R code, including results not presented in the main text.
(PDF)
