# Supplementary material for: Rapa Nui (Easter Island) monument (ahu) locations explained by freshwater sources
Source: PLoS One. 2019 Jan 10;14(1):e0210409. doi: 10.1371/journal.pone.0210409 (PMC6328247; doi:10.1371/journal.pone.0210409)
Supplement: S8 File — PDF file showing the output of running the R code, including results not presented in the main text. (PDF) [file pone.0210409.s008.pdf]

R code and supplementary results for - DiNapoli et al. (2018, in review with *PLOS ONE*) 'Rapa Nui (Easter Island) monument (*ahu*) locations explained by freshwater sources'

Robert J DiNapoli

December 18, 2018

LOAD PACKAGES

Load the necessary R packages.

```
library(spatstat) #for point process modeling
library(maptools) #for handling spatial data
library(raster) #for handling spatial data
library(rgdal) #for handling spatial data
library(sp) #for handling spatial data
library(MuMIn) #for multi-model selection
```

LOAD AND CONVERT DATA

Import necessary shapefiles and rasters. Ensure that data files are in the current working directory.

```
ahu <- readShapeSpatial("ahu_clipped.shp")

water <- readShapeSpatial("water_clipped.shp")

survey_area <- readShapeSpatial("survey_area_corrected_proj.shp")

rock_mulch_dist <- raster("mulch_dens_100_dist_clipped.tif")
rock_mulch_med_dist <- raster("mulch_dens_med_100_dist_clipped.tif")
rock_mulch_max_dist <- raster("mulch_dens_max_100_dist_clipped.tif")
coast_dist <- raster("coast_dist_clipped.tif")
marine_poly <- readShapeSpatial("marine_res_poly_s_10_clipped.shp")
```

Convert to fortmats interprettable by the spatstat package.

```
survey_win <- as.owin(survey_area)
ahu_pp <- ppp(ahu$POINT_X, ahu$POINT_Y, window=survey_win)
water_pp <- ppp(water$POINT_X, water$POINT_Y, window=survey_win)

## Warning: data contain duplicated points

water_pp <- unique(water_pp) #remove the small number of duplicates
mulch_dist <- as.im(rock_mulch_dist)
```

```
mulch_dist_med <- as.im(rock_mulch_med_dist)
mulch_dist_max <- as.im(rock_mulch_max_dist)
marine_res <- as.owin(marine_poly)
coast_dist <- as.im(coast_dist)
```

Create distance maps for freshwater and marine resources.

```
water_dist <- distmap(water_pp)
marine_dist <- as.im(distfun(marine_res), W=survey_win)
```

EXPLORATORY ANALYSES AND TESTS

Test of hypothesis 1. Perform L-function test and Inhomogeneous L-function test for ahu against the null hypothesis of Complete Spatial Randomness (CSR) with 39 simulations (equivalent to p=0.05). This code creates Fig 3.

```
ahu_L <- envelope(ahu_pp, fun=Lest, nsim=39, fix.n=T, global=T)

## Generating 39 simulations of CSR with fixed number of points  ...
## 1, 2, 3, 4, 5, 6, 7, 8, 9, 10, 11, 12, 13, 14, 15, 16, 17, 18, 19, 20, 21,
22, 23, 24, 25, 26, 27, 28, 29, 30, 31, 32, 33, 34, 35, 36, 37, 38,
##  39.
##
## Done.

ahu_Linhom <- envelope(ahu_pp, fun=Linhom, nsim=39, fix.n=T, global=T)

## Generating 39 simulations of CSR with fixed number of points  ...
## 1, 2, 3, 4, 5, 6, 7, 8, 9, 10, 11, 12, 13, 14, 15, 16, 17, 18, 19, 20, 21,
22, 23, 24, 25, 26, 27, 28, 29, 30, 31, 32, 33, 34, 35, 36, 37, 38,
##  39.
##
## Done.

#plot both, Fig 3.
par(mfrow=c(1,2))
plot(ahu_L, main="", xlab="r (meters)", ylim=c(0,3500), legend=F)
plot(ahu_Linhom, main="", xlab="r (meters)",ylim=c(0,3500), legend=F)

par(mfrow=c(1,1))
```

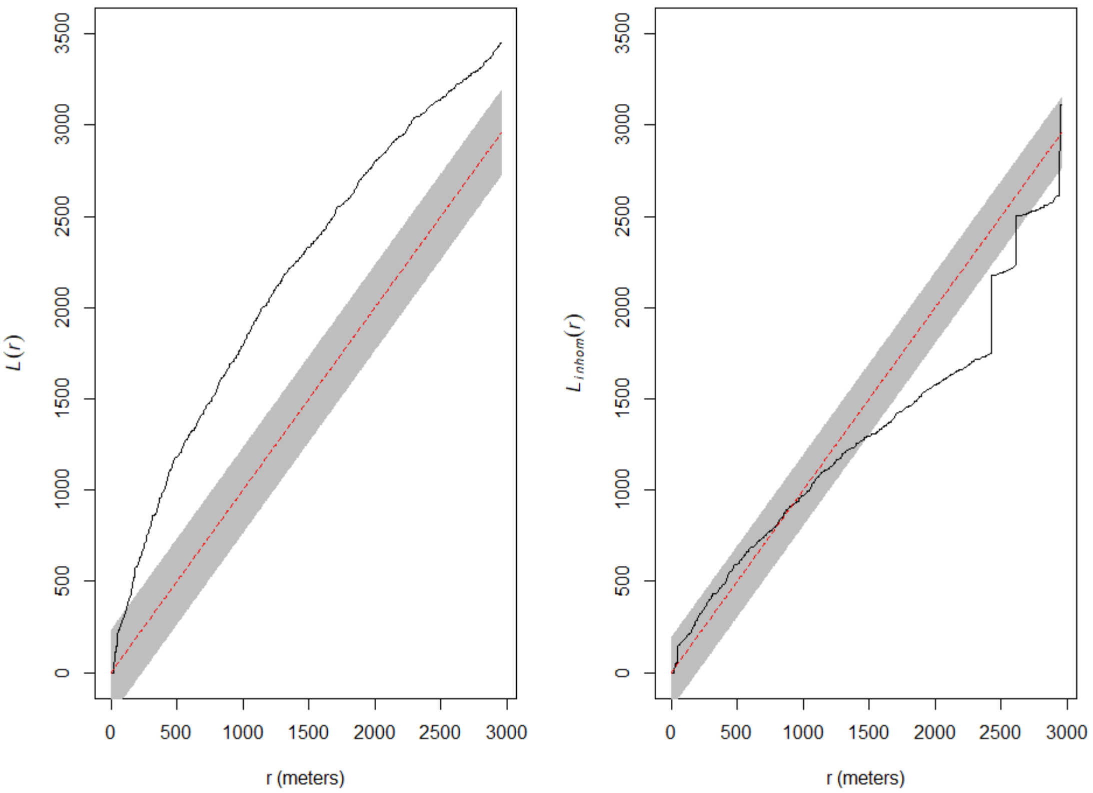

Perform spatial Kolmogorov-Smirnov tests for the relationship between ahu and distance from the minimal, medial, and maximimal rock mulch classifications, freshwater, and marine resources. Null hypothesis is CSR, alternative is that ahu are more closely spaced than random (i.e., "greater"). This code creates Fig 4 and includes results for the minimal and medial rock mulch classifications not presented in the main text.

```
#minimal rock mulch classification
ahu_mulch_cdf <- cdf.test(ahu_pp, mulch_dist, alternative="greater")
ahu_mulch_cdf

## 
##  Spatial Kolmogorov-Smirnov test of CSR in two dimensions
## 
## data:  covariate 'mulch_dist' evaluated at points of 'ahu_pp'
##      and transformed to uniform distribution under CSR
## D^+ = 0.082774, p-value = 0.2649
## alternative hypothesis: the CDF of x lies above the null hypothesis

plot(ahu_mulch_cdf, style="cdf", lwd=2, lwd0=2, do.legend=F,
     main="Spatial KS test of ahu & rock mulch", ylab="Probability", xlab="Di
stance to Rock Mulch")
```

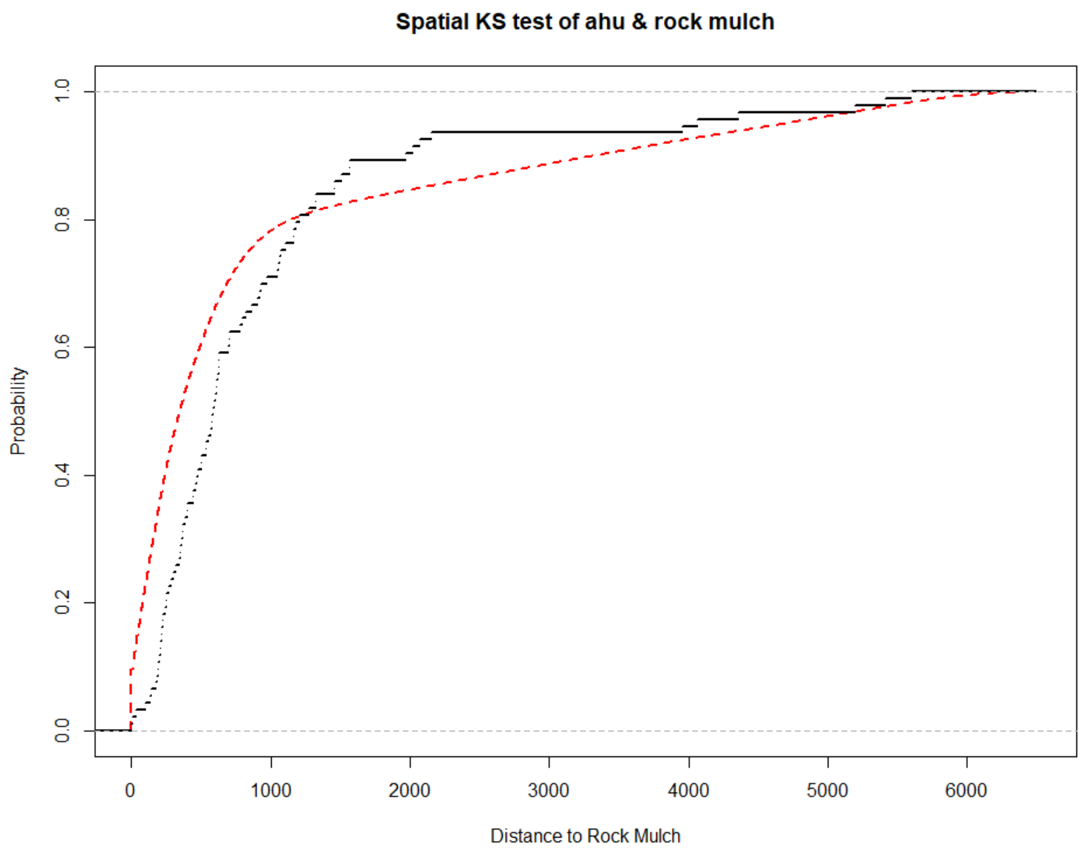


```
#medial rock mulch classification
ahu_mulch_med_cdf <- cdf.test(ahu_pp, mulch_dist_med, alternative="greater")
ahu_mulch_med_cdf

##
##  Spatial Kolmogorov-Smirnov test of CSR in two dimensions
##
## data:  covariate 'mulch_dist_med' evaluated at points of 'ahu_pp'
##      and transformed to uniform distribution under CSR
## D^+ = 0.20369, p-value = 0.0003677
## alternative hypothesis: the CDF of x lies above the null hypothesis

plot(ahu_mulch_med_cdf, style="cdf", lwd=2, lwd0=2, do.legend=F,
     main="Spatial KS test of ahu & rock mulch medial classification", ylab="
Probability", xlab="Distance to Rock Mulch")
```

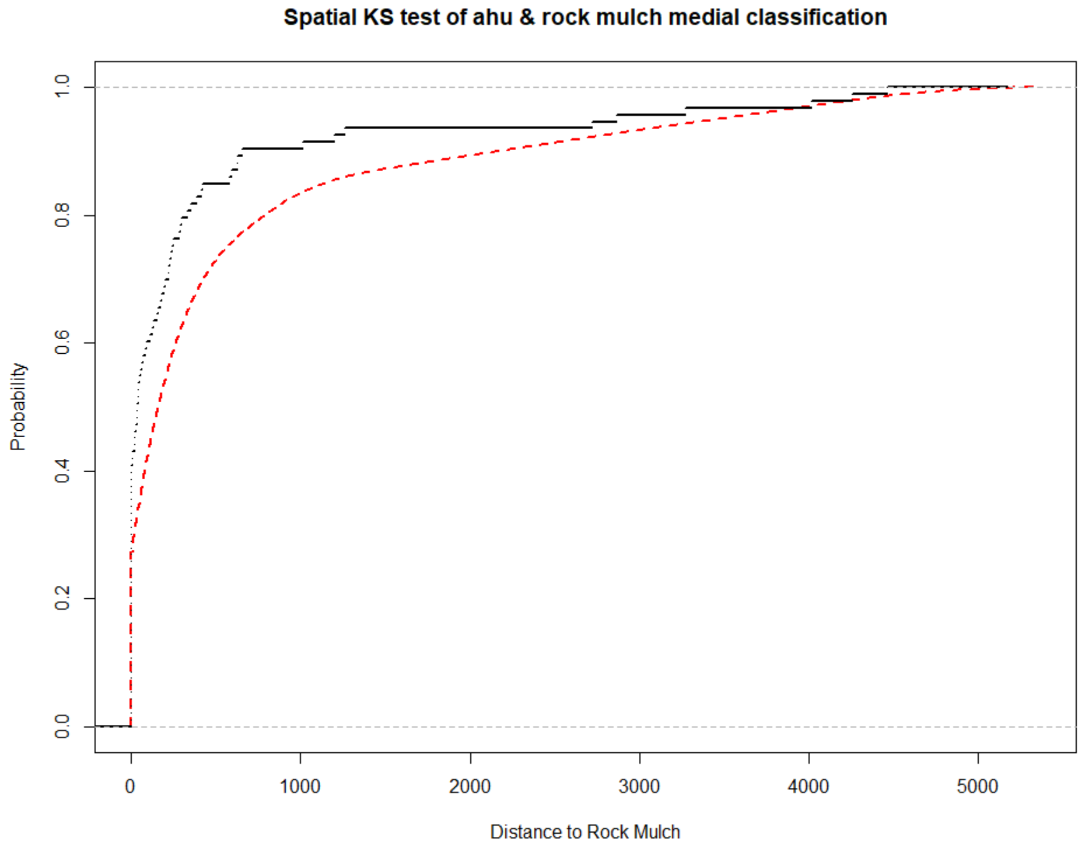


```
#maximal rock mulch classification
ahu_mulch_max_cdf <- cdf.test(ahu_pp, mulch_dist_max, alternative="greater")
ahu_mulch_max_cdf

##
##  Spatial Kolmogorov-Smirnov test of CSR in two dimensions
##
## data:  covariate 'mulch_dist_max' evaluated at points of 'ahu_pp'
##      and transformed to uniform distribution under CSR
## D^+ = 0.2413, p-value = 1.494e-05
## alternative hypothesis: the CDF of x lies above the null hypothesis

plot(ahu_mulch_max_cdf, style="cdf", lwd=2, lwd0=2, do.legend=F,
     main="Spatial KS test of ahu & rock mulch maximal classification", ylab=
"Probability", xlab="Distance to Rock Mulch")
```

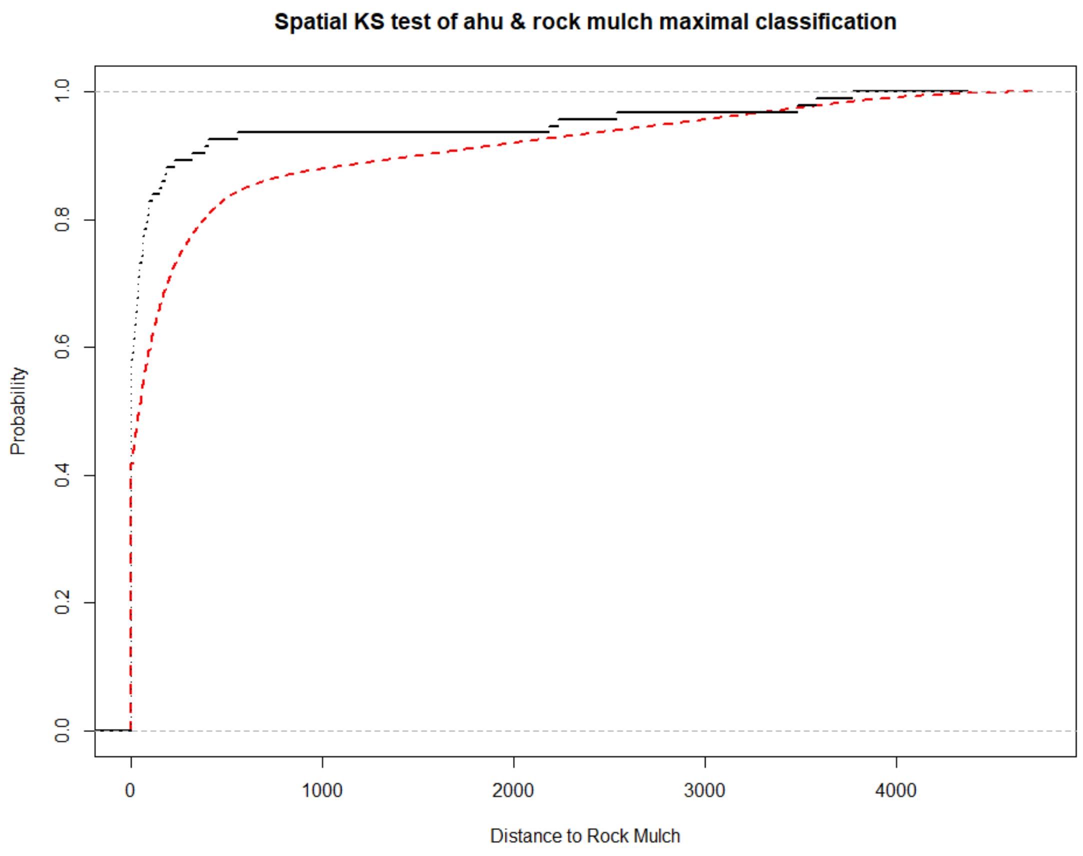


```
#freshwater
ahu_water_cdf <- cdf.test(ahu_pp, water_dist, alternative="greater")
ahu_water_cdf

##
##  Spatial Kolmogorov-Smirnov test of CSR in two dimensions
##
## data:  covariate 'water_dist' evaluated at points of 'ahu_pp'
##      and transformed to uniform distribution under CSR
## D^+ = 0.58982, p-value < 2.2e-16
## alternative hypothesis: the CDF of x lies above the null hypothesis

plot(ahu_water_cdf, style="cdf", lwd=2, lwd0=2, do.legend=F,
     main="SKS of ahu & freshwater", ylab="Probability", xlab="Distance to Fr
eshwater")
```

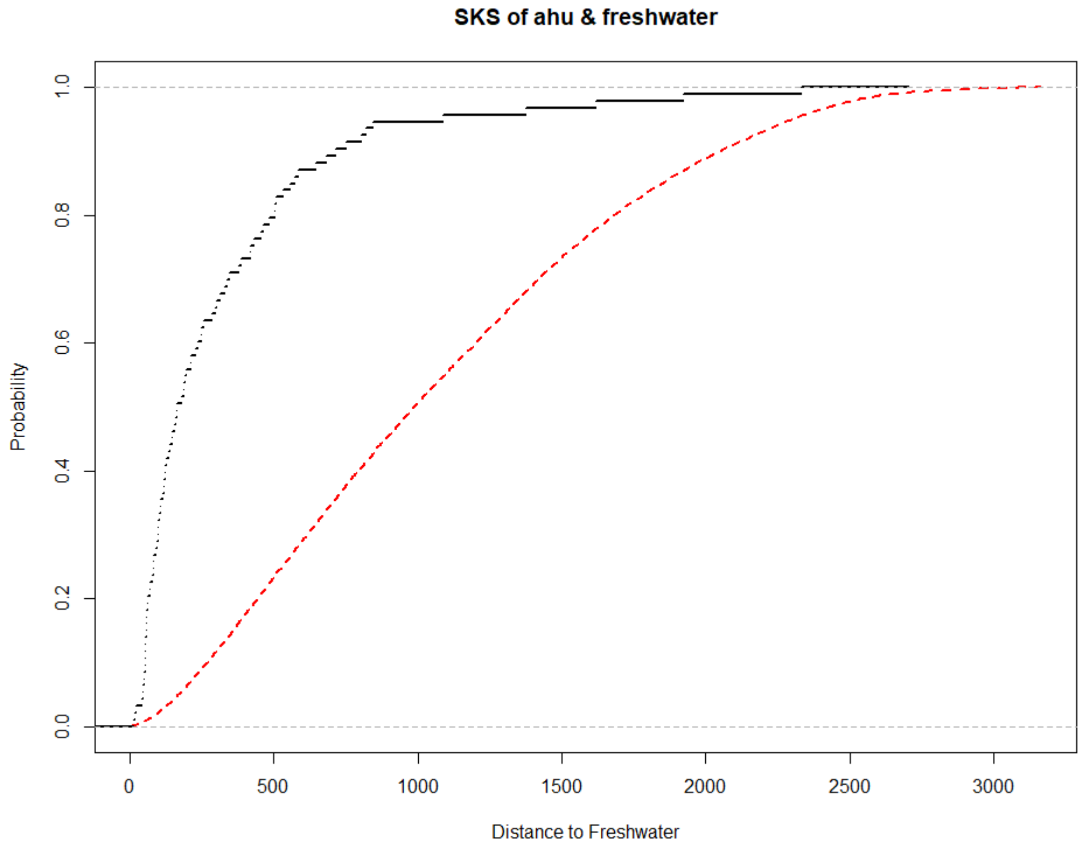


```
#marine resources
ahu_marine_cdf <- cdf.test(ahu_pp, marine_dist, alternative="greater")
ahu_marine_cdf

##
##  Spatial Kolmogorov-Smirnov test of CSR in two dimensions
##
## data:  covariate 'marine_dist' evaluated at points of 'ahu_pp'
##      and transformed to uniform distribution under CSR
## D^+ = 0.65478, p-value < 2.2e-16
## alternative hypothesis: the CDF of x lies above the null hypothesis

plot(ahu_marine_cdf, style="cdf", lwd=2, lwd0=2, do.legend=F,
     main="SKS of ahu & marine resources", ylab="Probability", xlab="Distance
to marine resources")
```

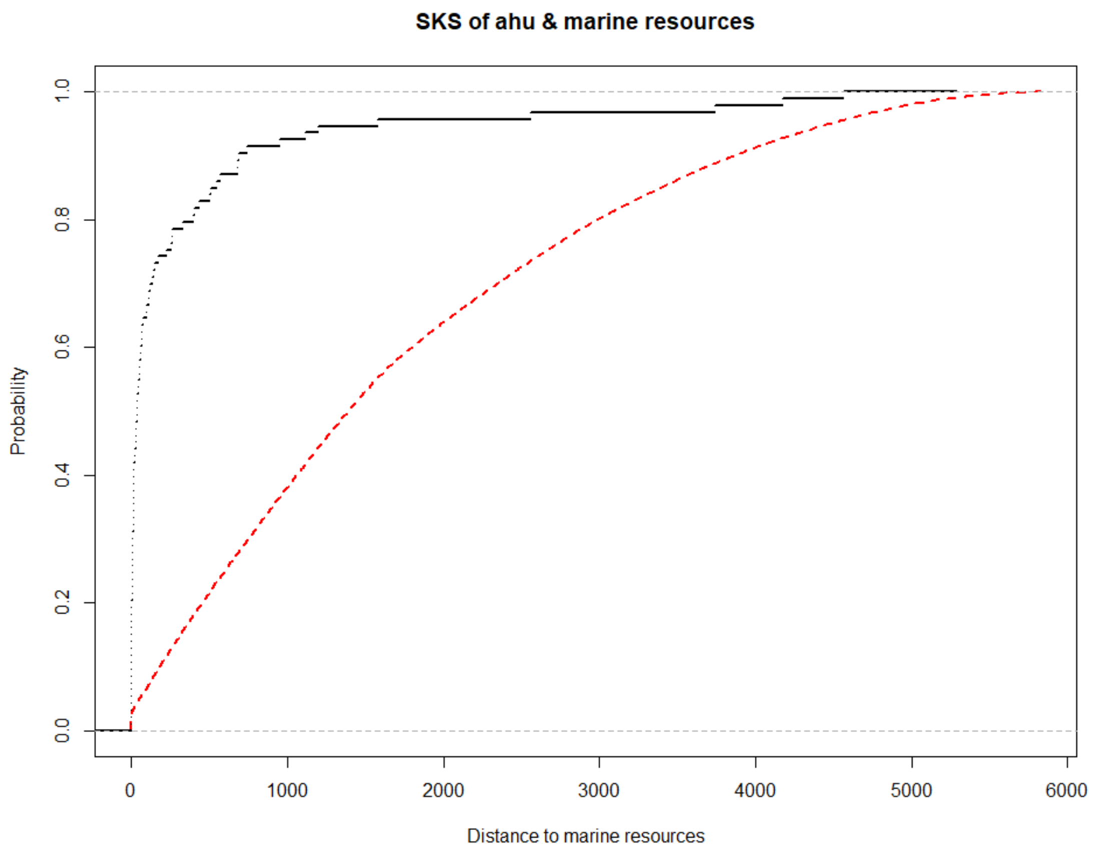


```
#plot together with Euclidean distance maps, Fig 4.
par(mfrow=c(3, 2))
plot(mulch_dist_max, main="Distance to rock mulch")
plot(ahu_pp, pch=15, add=T)
plot(ahu_mulch_max_cdf, style="cdf", lwd=2, lwd0=2, do.legend=F,
     main="SKS of ahu & rock mulch", ylab="Probability", xlab="Distance to ro
ck mulch")
plot(marine_dist, main="Distance to marine resources")
plot(ahu_pp, pch=15, add=T)
plot(ahu_marine_cdf, style="cdf", lwd=2, lwd0=2, do.legend=F,
     main="SKS of ahu & marine resources", ylab="Probability", xlab="Distance
to marine resources")
plot(water_dist, main="Distance to freshwater")
plot(ahu_pp, pch=15, add=T)
plot(ahu_water_cdf, style="cdf", lwd=2, lwd0=2, do.legend=F,
     main="SKS of ahu & freshwater", ylab="Probability", xlab="Distance to Fr
eshwater")

par(mfrow=c(1,1))
```

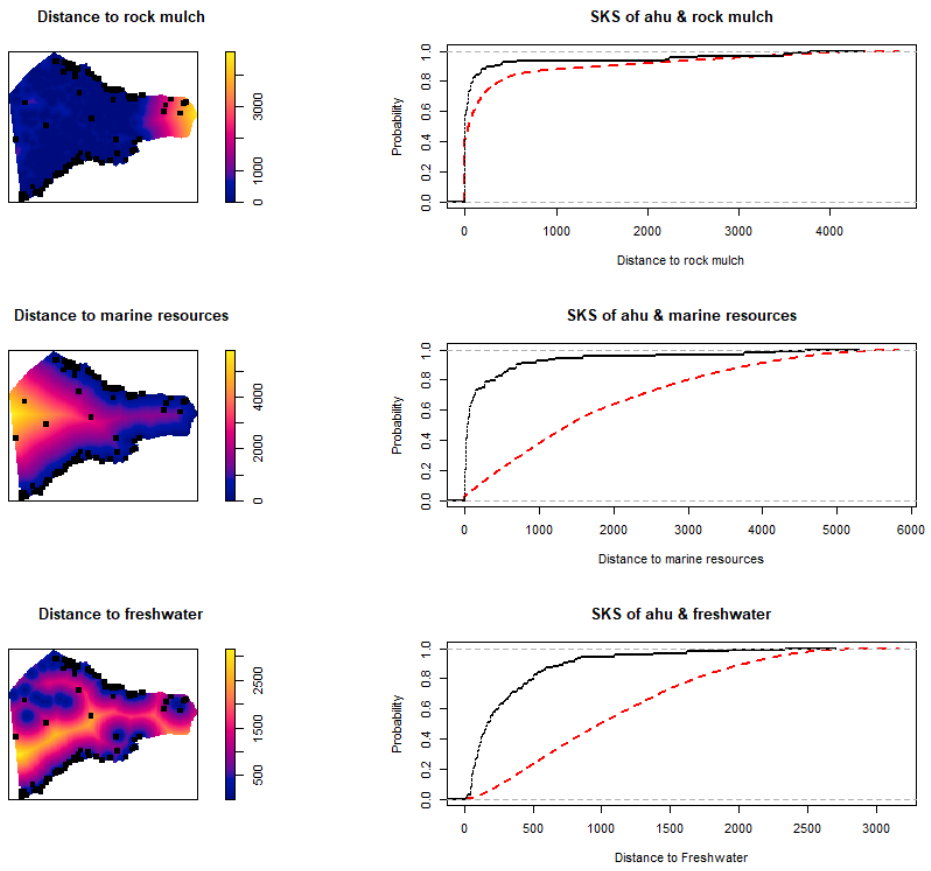

POINT PROCESS MODELS AND MULTIMODEL SELECTION

Build PPMS. See end of document for models with medial mulch classification.

```
model_1 <- ppm(ahu_pp, ~coast_dist)
model_2 <- ppm(ahu_pp, ~water_dist)
model_3 <- ppm(ahu_pp, ~marine_dist)
model_4 <- ppm(ahu_pp, ~mulch_dist_max)
model_5 <- ppm(ahu_pp, ~coast_dist+water_dist)
model_6 <- ppm(ahu_pp, ~coast_dist+marine_dist)
model_7 <- ppm(ahu_pp, ~coast_dist+mulch_dist_max)
model_8 <- ppm(ahu_pp, ~water_dist+marine_dist)
model_9 <- ppm(ahu_pp, ~water_dist+mulch_dist_max)
model_10 <- ppm(ahu_pp, ~marine_dist+mulch_dist_max)
model_11 <- ppm(ahu_pp, ~water_dist+marine_dist+mulch_dist_max)
model_12 <- ppm(ahu_pp, ~coast_dist+water_dist+marine_dist)
model_13 <- ppm(ahu_pp, ~coast_dist+water_dist+mulch_dist_max)
model_14 <- ppm(ahu_pp, ~coast_dist+marine_dist+mulch_dist_max)
model_15 <- ppm(ahu_pp, ~coast_dist+water_dist+marine_dist+mulch_dist_max)
```

Perform BIC ad AIC tests to choose model with parameter set that maximizes the tradeoff between model fit and complexity. Lowest delta-BIC/AIC and highest weight value indicate the best fitting model. This code creates Table 1.

```
MS_AIC <- model.sel(model_1, model_2, model_3, model_4, model_5, model_6, mod
el_7, model_8,
                    model_9, model_10, model_11, model_12, model_13, model_14
, model_15, rank=AIC)
MS_AIC

## Model selection table
##                          trend df    logLik    AIC  delta weight
## model_5                c_d+w_d  3 -1255.246 2516.5   0.00  0.350
## model_12          c_d+w_d+mr_d  4 -1254.310 2516.6   0.13  0.328
## model_15 c_d+w_d+mr_d+ml_d_mx  5 -1254.087 2518.2   1.68  0.151
## model_13      c_d+w_d+ml_d_mx  4 -1255.162 2518.3   1.83  0.140
## model_8               w_d+mr_d  3 -1257.999 2522.0   5.51  0.022
## model_11     w_d+mr_d+ml_d_mx  4 -1257.989 2524.0   7.49  0.008
## model_14     c_d+mr_d+ml_d_mx  4 -1270.418 2548.8  32.34  0.000
## model_7           c_d+ml_d_mx  3 -1272.887 2551.8  35.28  0.000
## model_10         mr_d+ml_d_mx  3 -1280.500 2567.0  50.51  0.000
## model_6               c_d+mr_d  3 -1281.200 2568.4  51.91  0.000
## model_1                    c_d  2 -1282.318 2568.6  52.14  0.000
## model_2                    w_d  2 -1284.824 2573.6  57.16  0.000
## model_9           w_d+ml_d_mx  3 -1284.716 2575.4  58.94  0.000
## model_3                   mr_d  2 -1288.190 2580.4  63.89  0.000
## model_4                ml_d_mx  2 -1373.108 2750.2 233.72  0.000
## Abbreviations:
## trend: c_d = '~coast_dist', c_d+mr_d = '~coast_dist+marine_dist',
##        c_d+mr_d+ml_d_mx = '~coast_dist+marine_dist+mulch_dist_max',
##        c_d+ml_d_mx = '~coast_dist+mulch_dist_max',
##        c_d+w_d = '~coast_dist+water_dist',
##        c_d+w_d+mr_d = '~coast_dist+water_dist+marine_dist',
##        c_d+w_d+mr_d+ml_d_mx = '~coast_dist+water_dist+marine_dist+mulch_di
st_max',
##        c_d+w_d+ml_d_mx = '~coast_dist+water_dist+mulch_dist_max',
##        mr_d = '~marine_dist',
##        mr_d+ml_d_mx = '~marine_dist+mulch_dist_max',
##        ml_d_mx = '~mulch_dist_max', w_d = '~water_dist',
##        w_d+mr_d = '~water_dist+marine_dist',
##        w_d+mr_d+ml_d_mx = '~water_dist+marine_dist+mulch_dist_max',
##        w_d+ml_d_mx = '~water_dist+mulch_dist_max'
## Models ranked by AIC(x)

MS_BIC <- model.sel(model_1, model_2, model_3, model_4, model_5, model_6, mod
el_7, model_8,
                    model_9, model_10, model_11, model_12, model_13, model_14
, model_15, rank=BIC)
MS_BIC
```

```
## Model selection table
##                        trend df    logLik    BIC  delta weight
## model_5               c_d+w_d  3 -1255.246 2524.1   0.00  0.675
## model_12         c_d+w_d+mr_d  4 -1254.310 2526.8   2.66  0.178
## model_13      c_d+w_d+ml_d_mx  4 -1255.162 2528.5   4.36  0.076
## model_8              w_d+mr_d  3 -1257.999 2529.6   5.51  0.043
## model_15 c_d+w_d+mr_d+ml_d_mx  5 -1254.087 2530.8   6.75  0.023
## model_11     w_d+mr_d+ml_d_mx  4 -1257.989 2534.1  10.02  0.005
## model_14     c_d+mr_d+ml_d_mx  4 -1270.418 2559.0  34.88  0.000
## model_7           c_d+ml_d_mx  3 -1272.887 2559.4  35.28  0.000
## model_1                   c_d  2 -1282.318 2573.7  49.61  0.000
## model_10         mr_d+ml_d_mx  3 -1280.500 2574.6  50.51  0.000
## model_6              c_d+mr_d  3 -1281.200 2576.0  51.91  0.000
## model_2                   w_d  2 -1284.824 2578.7  54.62  0.000
## model_9           w_d+ml_d_mx  3 -1284.716 2583.0  58.94  0.000
## model_3                  mr_d  2 -1288.190 2585.4  61.36  0.000
## model_4               ml_d_mx  2 -1373.108 2755.3 231.19  0.000
## Abbreviations:
## trend: c_d = '~coast_dist', c_d+mr_d = '~coast_dist+marine_dist',
##        c_d+mr_d+ml_d_mx = '~coast_dist+marine_dist+mulch_dist_max',
##        c_d+ml_d_mx = '~coast_dist+mulch_dist_max',
##        c_d+w_d = '~coast_dist+water_dist',
##        c_d+w_d+mr_d = '~coast_dist+water_dist+marine_dist',
##        c_d+w_d+mr_d+ml_d_mx = '~coast_dist+water_dist+marine_dist+mulch_di
st_max',
##        c_d+w_d+ml_d_mx = '~coast_dist+water_dist+mulch_dist_max',
##        mr_d = '~marine_dist',
##        mr_d+ml_d_mx = '~marine_dist+mulch_dist_max',
##        ml_d_mx = '~mulch_dist_max', w_d = '~water_dist',
##        w_d+mr_d = '~water_dist+marine_dist',
##        w_d+mr_d+ml_d_mx = '~water_dist+marine_dist+mulch_dist_max',
##        w_d+ml_d_mx = '~water_dist+mulch_dist_max'
## Models ranked by BIC(x)
```

Both AIC and BIC indicate model_5 is the best fitting model. Inspect model_5 coefficient estimates, standard errors, confidence intervals, and significance levels. This code creates Table 2.

```
model_5
```

```
## Nonstationary Poisson process
##
## Log intensity:  ~coast_dist + water_dist
##
## Fitted trend coefficients:
##   (Intercept)    coast_dist    water_dist
## -11.239422092  -0.001206134  -0.002534973
##
##                   Estimate         S.E.       CI95.lo       CI95.hi Ztest
## (Intercept) -11.239422092 0.1604265848 -11.553852420 -1.092499e+01   ***
```

```
## coast_dist   -0.001206134 0.0002560682  -0.001708018 -7.042493e-04   ***
## water_dist   -0.002534973 0.0004228176  -0.003363680 -1.706265e-03   ***
##                   Zval
## (Intercept) -70.059598
## coast_dist   -4.710205
## water_dist   -5.995428
```

Plot 'effect function' showing the relationship between the fitted intensity of ahu and distance from water sources. This code creates Fig 5.

```
plot(effectfun(model_5, "water_dist", coast_dist=0, se.fit=T), main="", xlab=
"Distance to freshwater (m)", ylab="Ahu intensity (λ)")
```

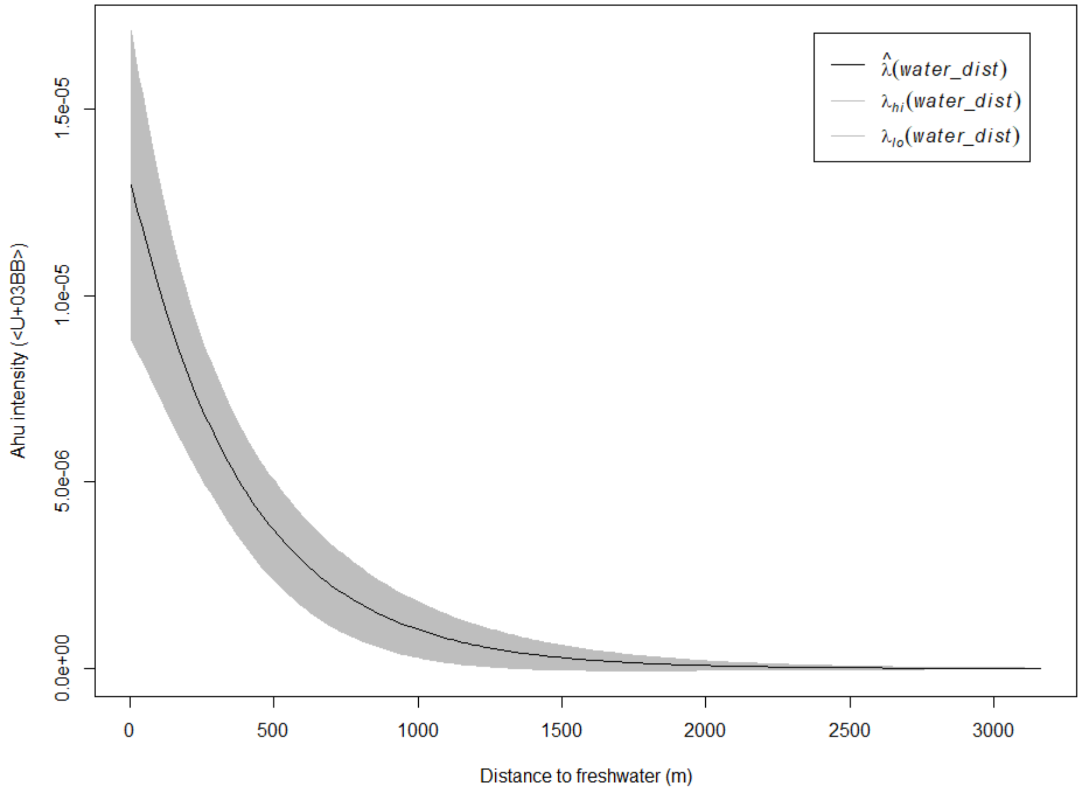

To further assess the fit of Model 5 and explore whether additional parameters are needed (such as second-order interpoint interaction), the following code executes a series of Monte Carlo-based goodness-of-fit tests: residual L-function (Manuscript Fig 6), maximum absolute deviation (MAD), and DCLF tests to assess the fit between model 2 and the ahu patterns. All Monte Carlo tests are ran with 39 simulated realizations of Model 5, which is equivalent to significance testing at p=0.05. Note that because these are randomized Monte Carlo tests, plots and/or resulting values may be slightly different than presented in the manuscript but the overall results will be the same.

```
L_fit1 <- envelope(model_5, Lest, nsim=39, global=T)
```

```
## Generating 78 simulated realisations of fitted Poisson model (39 to
## estimate the mean and 39 to calculate envelopes) ...
## 1, 2, 3, 4, 5, 6, 7, 8, 9, 10, 11, 12, 13, 14, 15, 16, 17, 18, 19, 20, 21,
22, 23, 24, 25, 26, 27, 28, 29, 30, 31, 32, 33, 34, 35, 36, 37, 38,
## 39, 40, 41, 42, 43, 44, 45, 46, 47, 48, 49, 50, 51, 52, 53, 54, 55, 56, 57
, 58, 59, 60, 61, 62, 63, 64, 65, 66, 67, 68, 69, 70, 71, 72, 73, 74, 75, 76,
## 77,  78.
##
## Done.
```

```
L_fit1
```

```
## Simultaneous critical envelopes for L(r)
## and observed value for 'model_5'
## Edge correction: "iso"
## Obtained from 39 simulations of fitted Poisson model
## Theoretical (i.e. null) mean value of L(r) estimated from a separate set
## of 39 simulations
## Alternative: two.sided
## Significance level of simultaneous Monte Carlo test: 1/40 = 0.025
## .............................................................
##       Math.label     Description
## r     r              distance argument r
## obs   hat(L)[obs](r) observed value of L(r) for data pattern
## mmean bar(L)(r)      sample mean of L(r) from simulations
## lo    hat(L)[lo](r)  lower critical boundary for L(r)
## hi    hat(L)[hi](r)  upper critical boundary for L(r)
## .............................................................
## Default plot formula:  .~r
## where "." stands for 'obs', 'mmean', 'hi', 'lo'
## Columns 'lo' and 'hi' will be plotted as shading (by default)
## Recommended range of argument r: [0, 2956.1]
## Available range of argument r: [0, 2956.1]
```

```
plot(L_fit1, main="", legend=F)
```

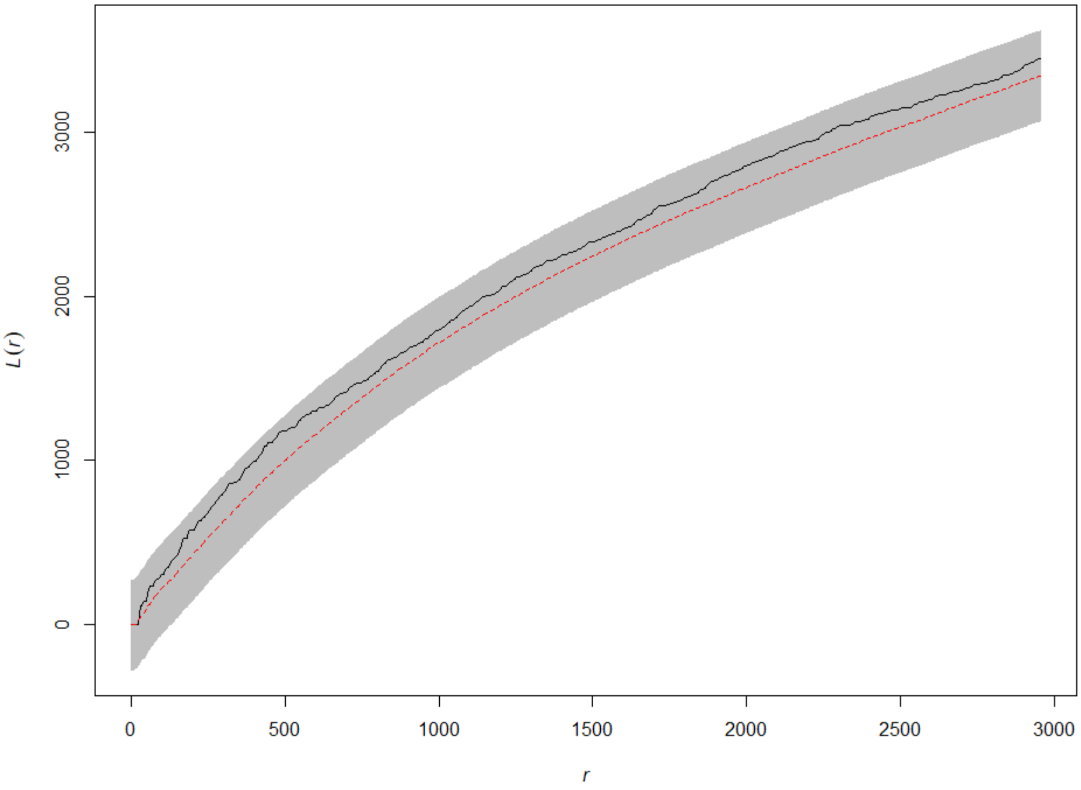


```
MAD1 <- mad.test(model_5, Lest, nsim=39, fix.n=T, global=T, alternative="two.
sided", use.theo=F)

## Generating 78 simulated realisations of fitted Poisson model with fixed
## number of points (39 to estimate the mean and 39 to calculate envelopes)
## ...
## 1, 2, 3, 4, 5, 6, 7, 8, 9, 10, 11, 12, 13, 14, 15, 16, 17, 18, 19, 20, 21,
22, 23, 24, 25, 26, 27, 28, 29, 30, 31, 32, 33, 34, 35, 36, 37, 38,
## 39, 40, 41, 42, 43, 44, 45, 46, 47, 48, 49, 50, 51, 52, 53, 54, 55, 56, 57
, 58, 59, 60, 61, 62, 63, 64, 65, 66, 67, 68, 69, 70, 71, 72, 73, 74, 75, 76,
## 77,  78.
##
## Done.

MAD1

##
##  Maximum absolute deviation test of fitted Poisson model
##  Monte Carlo test based on 78 simulations with fixed number of
##  points
##  Summary function: L(r)
##  Reference function: sample mean
##  Alternative: two.sided
##  Interval of distance values: [0, 2956.11269360199]
```

```
##  Test statistic: Maximum absolute deviation
##  Deviation = leave-one-out
##
## data:  model_5
## mad = 171.91, rank = 17, p-value = 0.2152
```

```
DCLF1 <- dclf.test(model_5, Lest, nsim=39, fix.n=T, global=T, alternative="tw
o.sided", use.theo=F)
```

```
## Generating 78 simulated realisations of fitted Poisson model with fixed
## number of points (39 to estimate the mean and 39 to calculate envelopes)
## ...
## 1, 2, 3, 4, 5, 6, 7, 8, 9, 10, 11, 12, 13, 14, 15, 16, 17, 18, 19, 20, 21,
22, 23, 24, 25, 26, 27, 28, 29, 30, 31, 32, 33, 34, 35, 36, 37, 38,
## 39, 40, 41, 42, 43, 44, 45, 46, 47, 48, 49, 50, 51, 52, 53, 54, 55, 56, 57
, 58, 59, 60, 61, 62, 63, 64, 65, 66, 67, 68, 69, 70, 71, 72, 73, 74, 75, 76,
## 77,  78.
##
## Done.
```

```
DCLF1
```

```
##
##  Diggle-Cressie-Loosmore-Ford test of fitted Poisson model
##  Monte Carlo test based on 78 simulations with fixed number of
##  points
##  Summary function: L(r)
##  Reference function: sample mean
##  Alternative: two.sided
##  Interval of distance values: [0, 2956.11269360199]
##  Test statistic: Integral of squared absolute deviation
##  Deviation = leave-one-out
##
## data:  model_5
## u = 41528000, rank = 14, p-value = 0.1772
```

Generate 20 simulated realization of the model. (Manuscript Fig 7). Note that the simulated realizations of the model will always appear slightly different than those presented in the manuscript (due to the functioning of the Metropolos-Hasting algortithm), though the overall results will be the same.

```
ahu_model_sim <- simulate(model_5, nsim=20)
```

```
## Generating 20 simulated patterns ...1, 2, 3, 4, 5, 6, 7, 8, 9, 10, 11, 12,
13, 14, 15, 16, 17, 18, 19,  20.
```

```
plot(ahu_model_sim, main="", pch=16)
```

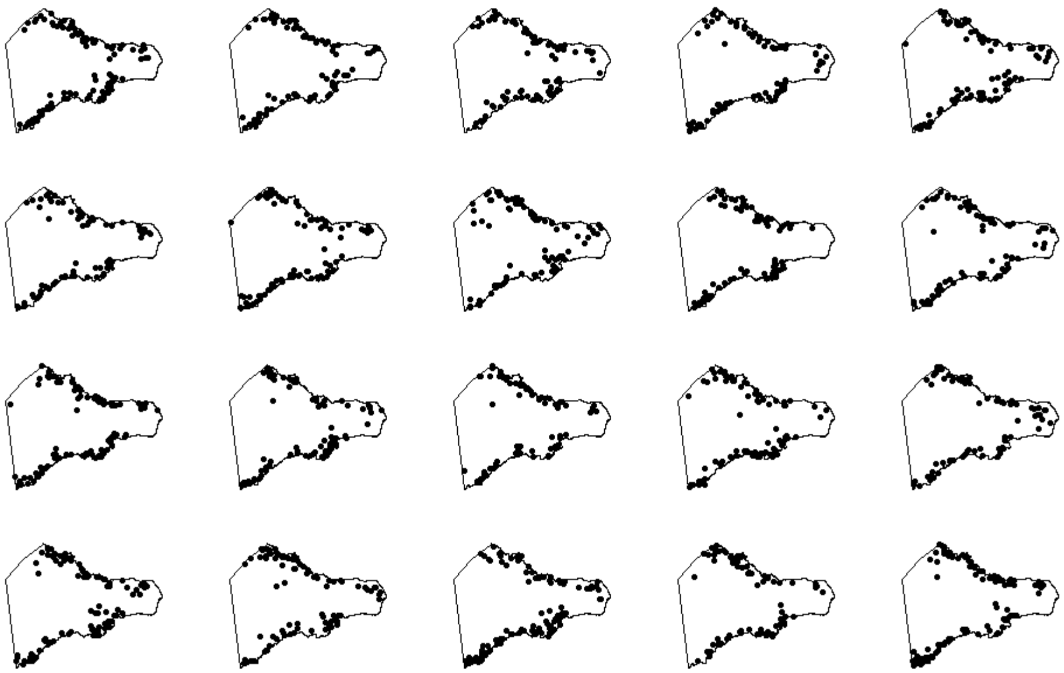

Models using the medial mulch classification from Ladefoged et al. 2013

```
med_model_1 <- ppm(ahu_pp, ~coast_dist)
med_model_2 <- ppm(ahu_pp, ~water_dist)
med_model_3 <- ppm(ahu_pp, ~marine_dist)
med_model_4 <- ppm(ahu_pp, ~mulch_dist_med)
med_model_5 <- ppm(ahu_pp, ~coast_dist+water_dist)
med_model_6 <- ppm(ahu_pp, ~coast_dist+marine_dist)
med_model_7 <- ppm(ahu_pp, ~coast_dist+mulch_dist_med)
med_model_8 <- ppm(ahu_pp, ~water_dist+marine_dist)
med_model_9 <- ppm(ahu_pp, ~water_dist+mulch_dist_med)
med_model_10 <- ppm(ahu_pp, ~marine_dist+mulch_dist_med)
med_model_11 <- ppm(ahu_pp, ~water_dist+marine_dist+mulch_dist_med)
med_model_12 <- ppm(ahu_pp, ~coast_dist+water_dist+marine_dist)
med_model_13 <- ppm(ahu_pp, ~coast_dist+water_dist+mulch_dist_med)
med_model_14 <- ppm(ahu_pp, ~coast_dist+marine_dist+mulch_dist_med)
med_model_15 <- ppm(ahu_pp, ~coast_dist+water_dist+marine_dist+mulch_dist_med
)
#model selection
med_MS_AIC <- model.sel(med_model_1, med_model_2, med_model_3, med_model_4, m
ed_model_5, med_model_6, med_model_7, med_model_8,med_model_9, med_model_10,
med_model_11, med_model_12, med_model_13, med_model_14, med_model_15, rank=AI
```

```
C)
med_MS_AIC
```

```
## Model selection table
##                              trend df    logLik    AIC  delta weight
## med_model_5                c_d+w_d  3 -1255.246 2516.5   0.00  0.345
## med_model_12          c_d+w_d+mr_d  4 -1254.310 2516.6   0.13  0.324
## med_model_15 c_d+w_d+mr_d+ml_d_md  5 -1254.026 2518.1   1.56  0.158
## med_model_13      c_d+w_d+ml_d_md  4 -1255.134 2518.3   1.78  0.142
## med_model_8               w_d+mr_d  3 -1257.999 2522.0   5.51  0.022
## med_model_11     w_d+mr_d+ml_d_md  4 -1257.980 2524.0   7.47  0.008
## med_model_14     c_d+mr_d+ml_d_md  4 -1269.712 2547.4  30.93  0.000
## med_model_7           c_d+ml_d_md  3 -1272.335 2550.7  34.18  0.000
## med_model_10         mr_d+ml_d_md  3 -1280.032 2566.1  49.57  0.000
## med_model_6              c_d+mr_d  3 -1281.200 2568.4  51.91  0.000
## med_model_1                   c_d  2 -1282.318 2568.6  52.14  0.000
## med_model_2                   w_d  2 -1284.824 2573.6  57.16  0.000
## med_model_9           w_d+ml_d_md  3 -1284.456 2574.9  58.42  0.000
## med_model_3                  mr_d  2 -1288.190 2580.4  63.89  0.000
## med_model_4               ml_d_md  2 -1372.625 2749.2 232.76  0.000
## Abbreviations:
## trend: c_d = '~coast_dist', c_d+mr_d = '~coast_dist+marine_dist',
##          c_d+mr_d+ml_d_md = '~coast_dist+marine_dist+mulch_dist_med',
##          c_d+ml_d_md = '~coast_dist+mulch_dist_med',
##          c_d+w_d = '~coast_dist+water_dist',
##          c_d+w_d+mr_d = '~coast_dist+water_dist+marine_dist',
##          c_d+w_d+mr_d+ml_d_md = '~coast_dist+water_dist+marine_dist+mulch_di
st_med',
##          c_d+w_d+ml_d_md = '~coast_dist+water_dist+mulch_dist_med',
##          mr_d = '~marine_dist',
##          mr_d+ml_d_md = '~marine_dist+mulch_dist_med',
##          ml_d_md = '~mulch_dist_med', w_d = '~water_dist',
##          w_d+mr_d = '~water_dist+marine_dist',
##          w_d+mr_d+ml_d_md = '~water_dist+marine_dist+mulch_dist_med',
##          w_d+ml_d_md = '~water_dist+mulch_dist_med'
## Models ranked by AIC(x)
```

```
med_MS_BIC <- model.sel(med_model_1, med_model_2, med_model_3, med_model_4, m
ed_model_5, med_model_6, med_model_7, med_model_8,med_model_9, med_model_10,
med_model_11, med_model_12, med_model_13, med_model_14, med_model_15, rank=BI
C)
med_MS_BIC
```

```
## Model selection table
##                              trend df    logLik    BIC  delta weight
## med_model_5                c_d+w_d  3 -1255.246 2524.1   0.00  0.672
## med_model_12          c_d+w_d+mr_d  4 -1254.310 2526.8   2.66  0.178
## med_model_13      c_d+w_d+ml_d_md  4 -1255.134 2528.4   4.31  0.078
## med_model_8               w_d+mr_d  3 -1257.999 2529.6   5.51  0.043
## med_model_15 c_d+w_d+mr_d+ml_d_md  5 -1254.026 2530.7   6.62  0.025
```

```
## med_model_11     w_d+mr_d+ml_d_md  4 -1257.980 2534.1  10.00  0.005
## med_model_14     c_d+mr_d+ml_d_md  4 -1269.712 2557.6  33.46  0.000
## med_model_7           c_d+ml_d_md  3 -1272.335 2558.3  34.18  0.000
## med_model_10         mr_d+ml_d_md  3 -1280.032 2573.7  49.57  0.000
## med_model_1                   c_d  2 -1282.318 2573.7  49.61  0.000
## med_model_6              c_d+mr_d  3 -1281.200 2576.0  51.91  0.000
## med_model_2                   w_d  2 -1284.824 2578.7  54.62  0.000
## med_model_9           w_d+ml_d_md  3 -1284.456 2582.5  58.42  0.000
## med_model_3                  mr_d  2 -1288.190 2585.4  61.36  0.000
## med_model_4               ml_d_md  2 -1372.625 2754.3 230.22  0.000
## Abbreviations:
## trend: c_d = '~coast_dist', c_d+mr_d = '~coast_dist+marine_dist',
##        c_d+mr_d+ml_d_md = '~coast_dist+marine_dist+mulch_dist_med',
##        c_d+ml_d_md = '~coast_dist+mulch_dist_med',
##        c_d+w_d = '~coast_dist+water_dist',
##        c_d+w_d+mr_d = '~coast_dist+water_dist+marine_dist',
##        c_d+w_d+mr_d+ml_d_md = '~coast_dist+water_dist+marine_dist+mulch_di
st_med',
##        c_d+w_d+ml_d_md = '~coast_dist+water_dist+mulch_dist_med',
##        mr_d = '~marine_dist',
##        mr_d+ml_d_md = '~marine_dist+mulch_dist_med',
##        ml_d_md = '~mulch_dist_med', w_d = '~water_dist',
##        w_d+mr_d = '~water_dist+marine_dist',
##        w_d+mr_d+ml_d_md = '~water_dist+marine_dist+mulch_dist_med',
##        w_d+ml_d_md = '~water_dist+mulch_dist_med'
## Models ranked by BIC(x)
```

Marine resources sensitivity analysis with a 5m threshold

```
marine_poly_5m <- readShapeSpatial("marine_res_poly_s_5_clipped.shp")

marine_res_5m <- as.owin(marine_poly_5m)
marine_dist_5m <- as.im(distfun(marine_res_5m), W=survey_win) #clip to survey
window

plot(marine_dist_5m)
```

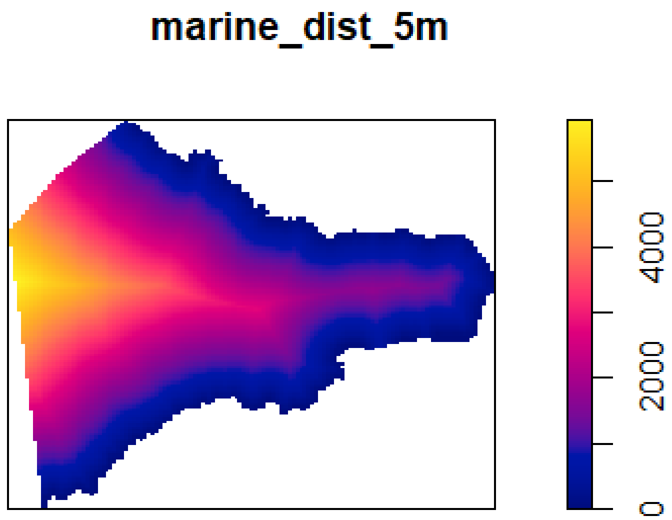


```
marine5m_model_1 <- ppm(ahu_pp, ~coast_dist)
marine5m_model_2 <- ppm(ahu_pp, ~water_dist)
marine5m_model_3 <- ppm(ahu_pp, ~marine_dist_5m)
marine5m_model_4 <- ppm(ahu_pp, ~mulch_dist_max)
marine5m_model_5 <- ppm(ahu_pp, ~coast_dist+water_dist)
marine5m_model_6 <- ppm(ahu_pp, ~coast_dist+marine_dist_5m)
marine5m_model_7 <- ppm(ahu_pp, ~coast_dist+mulch_dist_max)
marine5m_model_8 <- ppm(ahu_pp, ~water_dist+marine_dist_5m)
marine5m_model_9 <- ppm(ahu_pp, ~water_dist+mulch_dist_max)
marine5m_model_10 <- ppm(ahu_pp, ~marine_dist_5m+mulch_dist_max)
marine5m_model_11 <- ppm(ahu_pp, ~water_dist+marine_dist_5m+mulch_dist_max)
marine5m_model_12 <- ppm(ahu_pp, ~coast_dist+water_dist+marine_dist_5m)
marine5m_model_13 <- ppm(ahu_pp, ~coast_dist+water_dist+mulch_dist_max)
marine5m_model_14 <- ppm(ahu_pp, ~coast_dist+marine_dist_5m+mulch_dist_max)
marine5m_model_15 <- ppm(ahu_pp, ~coast_dist+water_dist+marine_dist_5m+mulch_
dist_max)


marine5m_MS_AIC <- model.sel(marine5m_model_1, marine5m_model_2, marine5m_mod
el_3, marine5m_model_4, marine5m_model_5,
                    marine5m_model_6, marine5m_model_7, marine5m_model_8, mar
ine5m_model_9, marine5m_model_10,
                    marine5m_model_11, marine5m_model_12, marine5m_model_13,
marine5m_model_14, marine5m_model_15, rank=AIC)
marine5m_MS_AIC
```

```
## Model selection table
##                                   trend df    logLik    AIC  delta weight
## marine5m_model_5                c_d+w_d  3 -1255.246 2516.5   0.00  0.393
## marine5m_model_8             w_d+mr_d_5  3 -1256.079 2518.2   1.67  0.171
## marine5m_model_13        c_d+w_d+ml_d_mx  4 -1255.162 2518.3   1.83  0.157
## marine5m_model_12         c_d+w_d+mr_d_5  4 -1255.198 2518.4   1.90  0.152
## marine5m_model_11     w_d+mr_d_5+ml_d_mx  4 -1256.043 2520.1   3.59  0.065
## marine5m_model_15 c_d+w_d+mr_d_5+ml_d_mx  5 -1255.096 2520.2   3.70  0.062
## marine5m_model_7            c_d+ml_d_mx  3 -1272.887 2551.8  35.28  0.000
## marine5m_model_14     c_d+mr_d_5+ml_d_mx  4 -1272.096 2552.2  35.70  0.000
## marine5m_model_10         mr_d_5+ml_d_mx  3 -1276.755 2559.5  43.02  0.000
## marine5m_model_1                    c_d  2 -1282.318 2568.6  52.14  0.000
## marine5m_model_6             c_d+mr_d_5  3 -1281.626 2569.3  52.76  0.000
## marine5m_model_2                    w_d  2 -1284.824 2573.6  57.16  0.000
## marine5m_model_9            w_d+ml_d_mx  3 -1284.716 2575.4  58.94  0.000
## marine5m_model_3                 mr_d_5  2 -1286.258 2576.5  60.02  0.000
## marine5m_model_4                ml_d_mx  2 -1373.108 2750.2 233.72  0.000
## Abbreviations:
## trend: c_d = '~coast_dist', c_d+mr_d_5 = '~coast_dist+marine_dist_5m',
##        c_d+mr_d_5+ml_d_mx = '~coast_dist+marine_dist_5m+mulch_dist_max',
##        c_d+ml_d_mx = '~coast_dist+mulch_dist_max',
##        c_d+w_d = '~coast_dist+water_dist',
##        c_d+w_d+mr_d_5 = '~coast_dist+water_dist+marine_dist_5m',
##        c_d+w_d+mr_d_5+ml_d_mx = '~coast_dist+water_dist+marine_dist_5m+mul
ch_dist_max',
##        c_d+w_d+ml_d_mx = '~coast_dist+water_dist+mulch_dist_max',
##        mr_d_5 = '~marine_dist_5m',
##        mr_d_5+ml_d_mx = '~marine_dist_5m+mulch_dist_max',
##        ml_d_mx = '~mulch_dist_max', w_d = '~water_dist',
##        w_d+mr_d_5 = '~water_dist+marine_dist_5m',
##        w_d+mr_d_5+ml_d_mx = '~water_dist+marine_dist_5m+mulch_dist_max',
##        w_d+ml_d_mx = '~water_dist+mulch_dist_max'
## Models ranked by AIC(x)
```

```
marine5m_MS_BIC <- model.sel(marine5m_model_1, marine5m_model_2, marine5m_mod
el_3, marine5m_model_4, marine5m_model_5,
                    marine5m_model_6, marine5m_model_7, marine5m_model_8, mar
ine5m_model_9, marine5m_model_10,
                    marine5m_model_11, marine5m_model_12, marine5m_model_13,
marine5m_model_14, marine5m_model_15, rank=BIC)
marine5m_MS_BIC
```

```
## Model selection table
##                                   trend df    logLik    BIC  delta weight
## marine5m_model_5                c_d+w_d  3 -1255.246 2524.1   0.00  0.583
## marine5m_model_8             w_d+mr_d_5  3 -1256.079 2525.8   1.67  0.253
## marine5m_model_13        c_d+w_d+ml_d_mx  4 -1255.162 2528.5   4.36  0.066
## marine5m_model_12         c_d+w_d+mr_d_5  4 -1255.198 2528.5   4.44  0.063
## marine5m_model_11     w_d+mr_d_5+ml_d_mx  4 -1256.043 2530.2   6.13  0.027
## marine5m_model_15 c_d+w_d+mr_d_5+ml_d_mx  5 -1255.096 2532.9   8.76  0.007
```

```
## marine5m_model_7             c_d+ml_d_mx  3 -1272.887 2559.4  35.28  0.000
## marine5m_model_14     c_d+mr_d_5+ml_d_mx  4 -1272.096 2562.3  38.23  0.000
## marine5m_model_10        mr_d_5+ml_d_mx  3 -1276.755 2567.1  43.02  0.000
## marine5m_model_1                     c_d  2 -1282.318 2573.7  49.61  0.000
## marine5m_model_6              c_d+mr_d_5  3 -1281.626 2576.9  52.76  0.000
## marine5m_model_2                     w_d  2 -1284.824 2578.7  54.62  0.000
## marine5m_model_3                  mr_d_5  2 -1286.258 2581.6  57.49  0.000
## marine5m_model_9             w_d+ml_d_mx  3 -1284.716 2583.0  58.94  0.000
## marine5m_model_4                 ml_d_mx  2 -1373.108 2755.3 231.19  0.000
## Abbreviations:
## trend: c_d = '~coast_dist', c_d+mr_d_5 = '~coast_dist+marine_dist_5m',
##        c_d+mr_d_5+ml_d_mx = '~coast_dist+marine_dist_5m+mulch_dist_max',
##        c_d+ml_d_mx = '~coast_dist+mulch_dist_max',
##        c_d+w_d = '~coast_dist+water_dist',
##        c_d+w_d+mr_d_5 = '~coast_dist+water_dist+marine_dist_5m',
##        c_d+w_d+mr_d_5+ml_d_mx = '~coast_dist+water_dist+marine_dist_5m+mul
ch_dist_max',
##        c_d+w_d+ml_d_mx = '~coast_dist+water_dist+mulch_dist_max',
##        mr_d_5 = '~marine_dist_5m',
##        mr_d_5+ml_d_mx = '~marine_dist_5m+mulch_dist_max',
##        ml_d_mx = '~mulch_dist_max', w_d = '~water_dist',
##        w_d+mr_d_5 = '~water_dist+marine_dist_5m',
##        w_d+mr_d_5+ml_d_mx = '~water_dist+marine_dist_5m+mulch_dist_max',
##        w_d+ml_d_mx = '~water_dist+mulch_dist_max'
## Models ranked by BIC(x)
```
